# Beyond speech perception: contemporary landscape of language agnostic hearing assessment

**DOI:** 10.3389/fnins.2026.1887850

**Published:** 2026-08-12

**Authors:** Archer Race Schaeffer, Yinglun Bi, William J. Bologna, Frederick J. Gallun, Tiffany Peng Hwa

**Affiliations:** 1 Department of Otolaryngology-Head and Neck Surgery, University of Pennsylvania Health System, Philadelphia, PA, United States; 2 Department of Speech-Language Pathology and Audiology, Towson University, Towson, MD, United States; 3 Oregon Hearing Research Center, Oregon Health and Science University, Portland, OR, United States

**Keywords:** auditory outcomes, cochlear implant, hearing impairment, language agnostic hearing assessment, spectral ripple discrimination, spectrotemporal, temporal modulation detection

## Abstract

Speech perception testing remains the dominant framework for cochlear implant (CI) outcomes assessment, yet its reliance on language introduces two fundamental limitations. Mechanistically, speech recognition performance reflects a composite of peripheral auditory encoding, cognitive processing, and linguistic knowledge, obscuring the contribution of each. Practically, language-dependent testing systematically underserves patients with limited English proficiency, prelingually deafened children, and others for whom verbal response formats are not viable. Language agnostic hearing tests (LAHTs) assess auditory function without requiring linguistic processing, offering a more precise probe of the auditory periphery and central encoding pathways and a more equitable framework for diverse populations. This narrative review was conducted via a systematic search of PubMed, MEDLINE, and Embase and examines the evidence for LAHTs across three domains: psychoacoustic resolution measures (spectral, temporal, and spectrotemporal), naturalistic stimulus measures (environmental sound identification, music perception, and phoneme discrimination), and electrophysiologic measures. Across the adult CI and hearing impairment literature, psychoacoustic measures demonstrate consistent, moderate-to-strong correlations with speech recognition outcomes. Spectral ripple discrimination and the Spectrotemporally Modulated Ripple Test (SMRT) predict speech outcomes in both English-speaking and non-English-speaking populations, though their validity at higher ripple densities is constrained by signal aliasing in CI processors. The Audible Contrast Threshold (ACT) has achieved clinical deployment for hearing aid fitting, and environmental sound identification deficits documented by the Familiar Environmental Sounds Test reveal safety-relevant dimensions of auditory function that speech batteries do not capture. In prelingually deafened children, the expected correlations between psychoacoustic resolution and speech recognition are largely absent, suggesting qualitatively distinct auditory reorganization that current measures incompletely characterize. Across domains, LAHT-speech correlations are real but partial: these measures predict speech outcomes without replacing them, and their relationship to patient-reported quality of life remains uninvestigated. The evidence supports integrating LAHTs as complementary tools in clinical practice and research, with the greatest immediate utility in candidacy screening, outcomes characterization in linguistically diverse populations, and mechanistic investigation of device and patient factors underlying outcome variability.

## 1 Introduction

The sustained development in cochlear implant (CI) candidacy criteria, hearing aid (HA) fitting, and improvements in device technology have been accompanied by a parallel evolution in outcome measurements, yet the dominant framework for assessing hearing function remains largely unchanged: pure-tone audiometry, word and sentence recognition tests administered in quiet and in noise, conducted in the language of the clinician and the test materials. This reliance of speech testing on language introduces fundamental limitations in the assessment of auditory function. Two such limitations are particularly salient: one neurocognitive, the other sociolinguistic.

**FIGURE 1:**
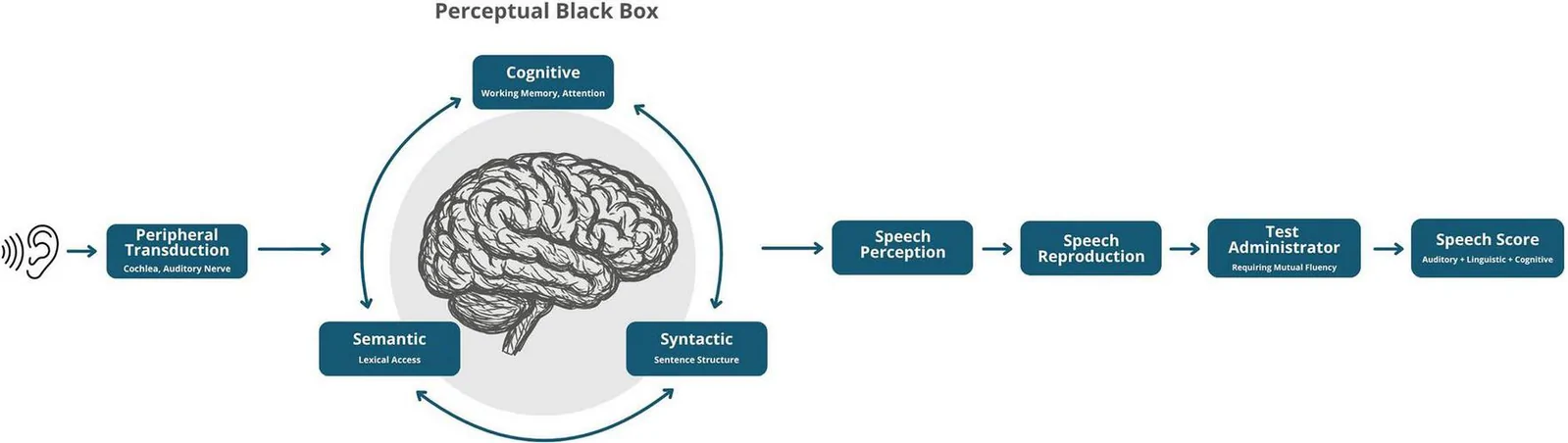
The blackbox of auditory perception. Standard speech recognition scores reflect not only peripheral auditory resolution but also the cumulative influence of cognitive load, semantic and syntactic knowledge, speech reproduction demands, and test administrator reporting, none of which are direct indices of auditory function.

Hearing and language processing are related yet distinct processes that introduce fundamental biases when speech perception tests are used to assess auditory function alone. Hearing is primarily a bottom-up process in which acoustic stimuli are transduced by the peripheral auditory system and transmitted via afferent cochlear pathways to central auditory structures. Language perception, by contrast, is predominantly top-down, in which higher-order cortical networks construct semantic, lexical, and syntactic structure from signals that do not intrinsically possess meaning. Speech perception emerges from the interaction of these two processes, creating an auditory black box in which test scores reflect a composite of auditory, linguistic, and cognitive functioning rather than hearing ability alone (Figure 1; Messersmith et al., 2019). This relationship between cognitive ability and speech perception strengthens in parallel with the cognitive demands of the task. In one study, no neurocognitive measure was associated with AzBio sentence recognition in quiet, whereas in multitalker babble working memory was the dominant predictor, accounting for 66% of the variance in performance [*F*(1, 14) = 11.65, *p* = 0.002] (Moberly et al., 2018a). It appears that as speech tasks approach the conditions of everyday listening, progressing from words in quiet to sentences in quiet to sentences in noise, they appear to draw increasingly on cognitive ability rather than auditory function alone.

The clinical consequences of this cognitive entanglement are substantial. Patients with similar preoperative characteristics who undergo technically comparable surgeries can experience widely divergent speech perception outcomes (Dornhoffer et al., 2021), and speech measures have not been able to resolve this ambiguity. Moreover, even when speech recognition performance is similar across patients, such measures poorly predict patient-reported benefits. McRackan et al. (2018) demonstrated that speech recognition scores in quiet and in noise explain less than 6% of the variance in post-implantation quality of life.

Beyond these neurocognitive constraints, reliance on language-based testing introduces a substantial and growing social limitation in an increasingly linguistically diverse United States, a country in which approximately 10% of the U.S. workforce has limited English proficiency (LEP) (Batalova and Zong, 2016). For people with LEP, language-dependent speech testing often fails to accurately reflect auditory function, yet such scores often serve as necessary criteria for insurance coverage and CI candidacy. One survey found that approximately 25% of US clinicians reported foregoing speech testing entirely for patients who speak neither English nor Spanish (Govindan et al., 2025). Breakdowns in cross-linguistic communication erode the provider’s ability to confidently evaluate patients with LEP, contributing to reduced CI referral rates despite these patients being no less likely to proceed with implantation once deemed candidates (Greiner et al., 2023). Although validated AzBio materials exist in several non-English languages (French, Spanish, Hebrew, and Mandarin), their use requires certified medical interpreters or mutual clinician-patient fluency (Bergeron et al., 2019; Rivas et al., 2021; Taitelbaum-Swead et al., 2022; Xi et al., 2022). Additionally, dialectal variability (e.g., a Spanish speaker from Colombia versus Cuba) within a given language can influence speech perception scores (Rodriguez and Shader, 2025). These challenges in clinical utilization are reflected in the literature: as of 2025, only three published studies used the Hebrew (HeBio) or Chinese Mandarin (CMnBio) sentence lists to report postoperative outcomes, representing a combined total of 85 patients across the two languages (Jung et al., 2026).

**FIGURE 2:**
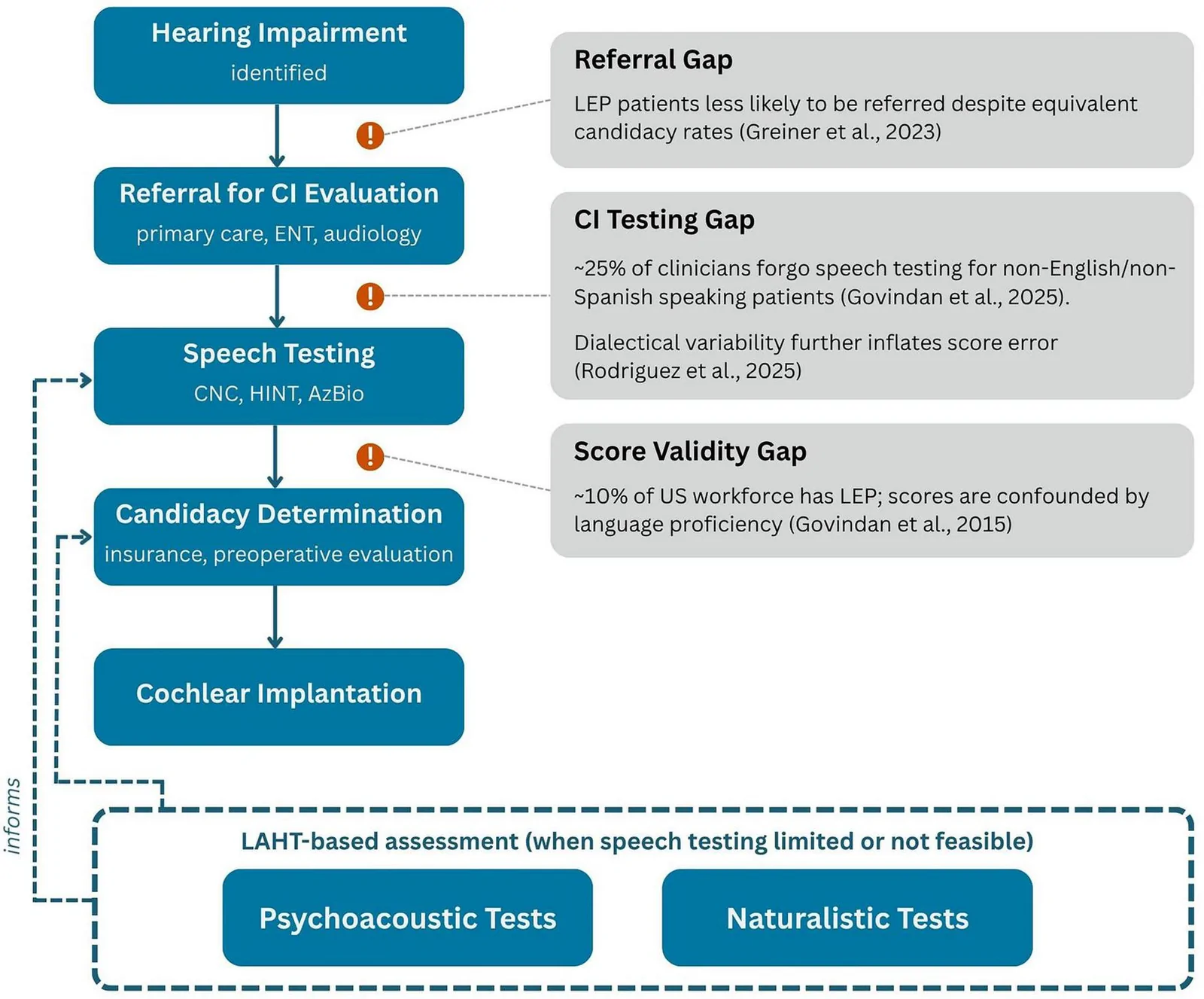
Language-dependent assessment and the equity gap in cochlear implant access. The figure maps the clinical pipeline from hearing loss identification through cochlear implantation surgery, identifying the stages at which language-dependent speech testing introduces systematic barriers for patients with limited English proficiency and other linguistically diverse populations. Attrition at these stages contributes to documented disparities in CI referral and candidacy rates that are not attributable to differences in audiometric profile.

These limitations affect not only the CI population but also the broader hearing-impaired population. Among hearing aid (HA) users, standard audiograms do not capture the supra-threshold distortion that underlies persistent speech-in-noise difficulties despite adequate amplification (Plomp, 1986; Sanchez-Lopez et al., 2020). The standard measures that guide the majority of clinical fitting decisions (e.g., pure-tone average, speech-in-quiet) demonstrate poor predictive power for aided speech-in-noise performance, and the language-based measures are subject to the same confounds as mentioned above (Duquesnoy, 1983; Grant and Walden, 2013; Killion et al., 2004; Killion and Niquette, 2000; Nilsson et al., 1994; Smoorenburg, 1992). The result is an equity gap embedded in standard clinical practice across all hearing impaired (HI) populations (Figure 2).

This work examines the role of language agnostic hearing tests (LAHTs) in the audiological assessment of individuals with hearing loss, with primary emphasis on their application in cochlear implant candidacy and outcomes evaluation. This is a natural extension of the field’s longstanding commitment to refining outcome measures in step with technological and demographic change. From the early adoption of the BKB and HINT sentence tests through the development of the Minimum Speech Test Battery (MSTB), testing paradigms have iteratively evolved to address ceiling effects and differentiate performance across a wider range of listeners and listening conditions (Shafiro et al., 2025). LAHTs continue this trajectory by characterizing auditory function without relying on linguistic processing, thereby mitigating confounding neurocognitive and sociolinguistic factors.

Although these measures may appear less ecologically valid than speech-in-noise formats, they offer distinct advantages: a more precise probe of the relationship between auditory physiology and language perception and an equitable assessment across linguistically diverse populations (Archer-Boyd et al., 2018; Moberly et al., 2018b; O’Neill et al., 2019). In contrast to findings by Moberly et al. (2018a) which show that cognitive ability can confound speech scores, O’Neill et al. (2019) demonstrated that working memory correlates significantly with speech recognition in CI users but not with spectral resolution performance, suggesting that this psychoacoustic measure can capture auditory function independent of cognitive load. Hearing metrics such as these have been recognized by the National Academies as an important direction for future research and show promise across clinical and laboratory settings in multiple countries and languages (Commitee on Meaningful Outcome Measures in Adult Hearing Health Care, 2025; Jeddi et al., 2019; van Groesen et al., 2023). This potential, however, must be weighed against the maturity of the evidence. No LAHT reviewed here has been validated sufficiently to replace speech testing, nor is replacement the goal. Until their predictive relationships, psychometric properties, and associations with patient-reported outcomes are sufficiently characterized, these measures will continue to occupy an undefined and underutilized role in hearing healthcare, even as they probe dimensions of auditory function that speech testing alone cannot reach.

In this paper, we examine the current landscape of LAHTs and compare their benefits and challenges to current practices. The types of LAHTs span two main categories: psychoacoustic resolution measures (spectral, temporal, and spectrotemporal) and naturalistic stimulus measures (environmental sound identification, music enjoyment, and phoneme perception), with additional coverage of electrophysiologic measures, outlined in Table 1. For each category, we consider the evidence supporting its relationship to speech outcomes, its clinical feasibility, and its limitations, beginning with spectral resolution.

**TABLE 1:** Summary of language agnostic hearing tests (LAHTs).

Domain	Sub-domain	Test	Publication (author, year)		Sample type and size (CI/HI/NH; n)
Psycho-acoustic resolution	Spectral	SRD	Henry and Turner (2003)		CI; *n* = 21 NH; *n* = 8
			Won et al. (2007)		CI; *n* = 31
			Anderson et al. (2011)		CI; *n* = 15
			Drennan et al. (2014)		CI; *n* = 28
			Shim et al. (2014)		HI; *n* = 15
			Dai et al. (2018)		CI; *n* = 17 NH; *n* = 23
		QSMD/EasyQSMD	Gifford et al. (2014)		CI; *n* = 76
			Gifford et al. (2018)		CI; *n* = 578
	Temporal	MDT/EasyMDT	Won et al. (2011)		CI; *n* = 24
			Shim et al. (2014)		CI; *n* = 28
			Landsberger et al. (2019a)		CI; *n* = 20
			Landsberger and Stupak (2022)		CI; *n* = 12 NH; *n* = 10
	Spectro-temporal	SMRT	Aronoff and Landsberger (2013)		NH; *n* = 8
			Holden et al. (2016)		CI; *n* = 114
			Lawler et al. (2017)		CI; *n* = 25
			Goehring et al. (2019)		CI; *n* = 8
			Jeddi et al. (2019)		CI; *n* = 75
			van Groesen et al. (2023)		CI; *n* = 28
	Spectro-temporal	STRIPES	Archer-Boyd et al. (2018)		CI; *n* = 8 NH; *n* = 8
			Archer-Boyd et al. (2020)		CI; *n* = 10
			Goehring et al. (2019)		CI; *n* = 8
			van Groesen et al. (2023)		CI; *n* = 28
		STM Detection	Choi et al. (2016)		HI; *n* = 27
		ACT	Zaar et al. (2023)		HI; *n* = 13
			Zaar et al. (2024a)		HI; *n* = 30
			Zaar et al. (2024b)		HI; *n* = 28
Naturalistic testing	Phoneme/Logatome	Logatome	Rahne et al. (2010)		CI; *n* = 8 NH; *n* = 13
		LIT-AD	Ching et al. (2021)		CI; *n* = 40 HA; *n* = 41
	Environmental sounds	FEST-I	Shafiro (2008a)		NH; *n* = 21
			Shafiro et al. (2011)		CI; *n* = 17
			Harris et al. (2017)		CI; *n* = 35 NH; *n* = 41
	Music experience	CAMP/Music	Nimmons et al. (2008)		CI; *n* = 8
Electrophysiology	Subcortical	ABR/EABR	N/A		
		ASSR			
	Cortical	CAEP			
		MMN			
		P300			
		E-ACT	Simonsen et al. (2025)	NH; *n* = 18 and 47	
			Simonsen et al. (2026)	NH; *n* = 18 and 47	

Tests are organized by domain and sub-domain. For each test, representative publications and sample characteristics are provided. Sample type denotes cochlear implant users (CI), listeners with hearing-impairment (HI), hearing aid users (HA), and normal-hearing controls (NH); sample size (n) reflects the number of participants in the cited study. Where electrophysiologic measures are listed without a specific publication, the measures are presented as clinical tools for which no single defining validation study in the CI or HA population is identified in this review. ABR, auditory brainstem response; ACT, Audible Contrast Threshold; ASSR, auditory steady-state response; CAEP, cortical auditory evoked potential; CAMP, Cochlear Implant Music Perception test; EABR, electrically evoked auditory brainstem response; FEST-I, Familiar Environmental Sounds Test – Identification; LIT-AD, Language-Independent Test of Auditory Discrimination; MDT, modulation detection threshold; MMN, mismatch negativity; QSMD, quick spectral modulation detection; SMRT, Spectrotemporally Modulated Ripple Test; SRD, spectral ripple discrimination; STM, spectrotemporal modulation; STRIPES, Spectro-Temporal Ripple for Investigating Processor Effectiveness.

### 1.1 Methods

This manuscript presents a narrative review of LAHTs for cochlear implant and hearing-impaired populations, conducted with a systematic search strategy outlined in the JBI Manual for Evidence Synthesis. A preliminary PubMed search was used to identify relevant text words, which informed the construction of concept blocks linked by Boolean operators targeting four domains: auditory and hearing test context; spectral, temporal, and spectrotemporal test instruments by full name or abbreviation; language agnostic as a construct; and cochlear implantation as the primary study population. (The full search strings are provided in Supplementary Appendix 1) The search was executed across PubMed, MEDLINE via Ovid, and Embase on November 30, 2025, with no date restrictions and an English-language filter applied.

**FIGURE 3:**
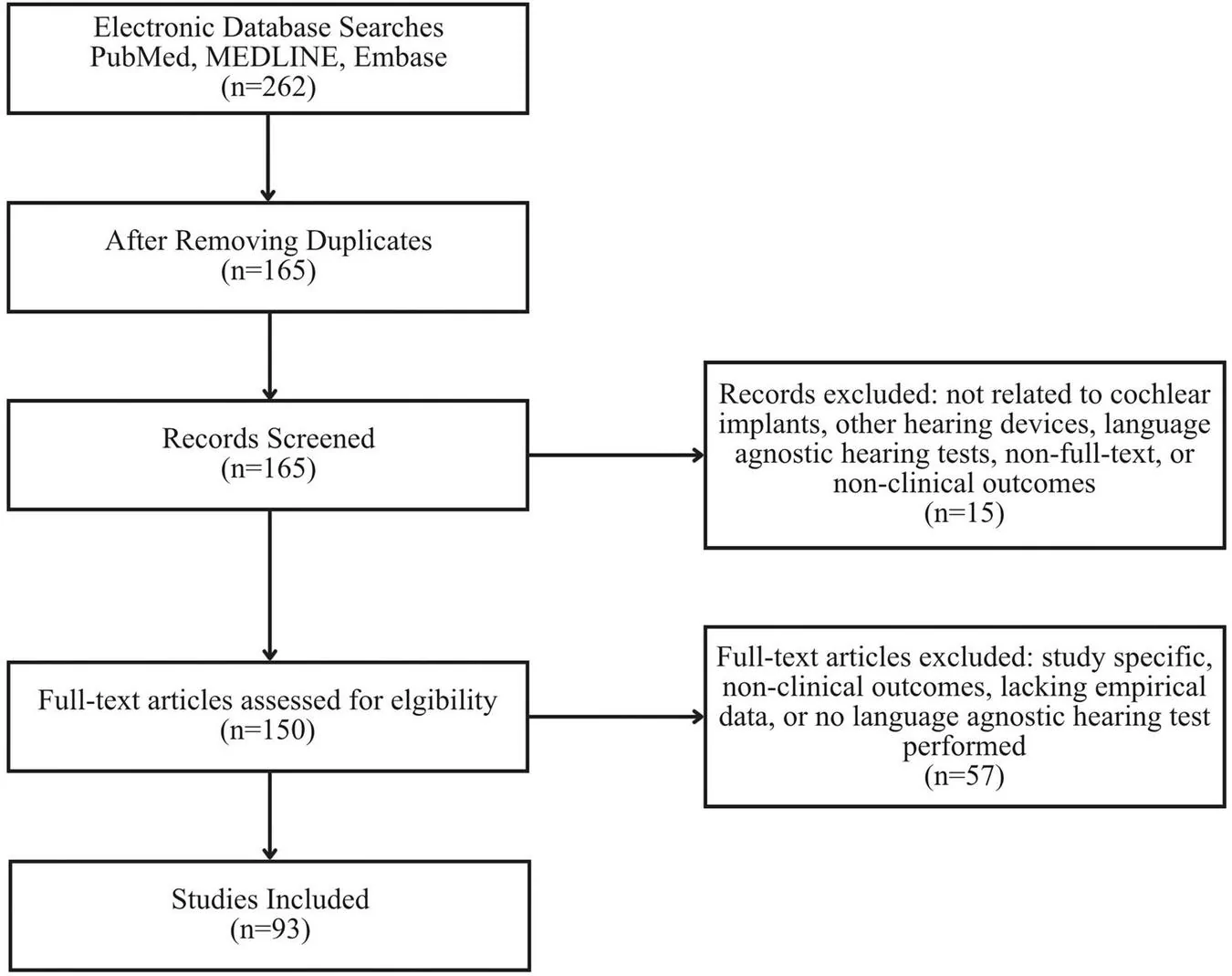
PRISMA diagram

All records were imported into EndNote, deduplicated, and screened from the final deduplicated reference library. Title and abstract screening was performed by a single reviewer. Studies were included if they involved human participants with sensorineural hearing loss using cochlear implants or hearing aids, administered at least one language agnostic or non-linguistic hearing measure, and were published as peer-reviewed original research or review articles in English. Full-text retrieval was reserved for studies identified through citation tracking using Zotero (version 9.0.3). Record flow is summarized in Figure 3.

## 2 Psychoacoustic resolution

The measures examined in this section are unified by their stimuli: synthetic, non-linguistic signals devoid of the lexical and semantic content of speech. By minimizing the contribution of linguistic knowledge, these tests serve the paper’s central aim of distinguishing what a patient can hear from what they can understand.

### 2.1 Spectral resolution tests

Spectral resolution, the auditory system’s capacity to resolve distinct frequency components within a complex sound, represents a fundamental domain of language agnostic hearing assessment. In the normal-hearing cochlea, thousands of hair cells provide fine tonotopic resolution, enabling the discrete perception of closely spaced frequency components of a sound. In CI users, this biological architecture is replaced by a small array of electrodes, typically 12 to 22, each responsible for encoding a broad band of acoustic frequencies and subject to spread of excitation that further reduces the specificity of individual channels. This technologic constraint imposes inherent limits on spectral resolution that pure-tone thresholds alone cannot reveal, motivating the development of psychoacoustic tools specifically designed to quantify spectral resolution.

Three principal approaches to quantifying spectral resolution are reviewed below: spectral ripple discrimination (SRD), which tracks the highest ripple density at which listeners detect spectral phase reversals; spectral modulation detection (SMD) and its clinical derivative the Quick Spectral Modulation Detection (QSMD), which measure the minimum modulation depth required to detect spectral structure; and spatial tuning curves, which index electrode selectivity via forward-masked electrical stimulation. SRD and SMD have been studied across both CI and HA populations, whereas special tuning curves are restricted to CI users, as they examine electrode-level channel selectivity.

#### 2.1.1 Spectral ripple discrimination

**FIGURE 4:**
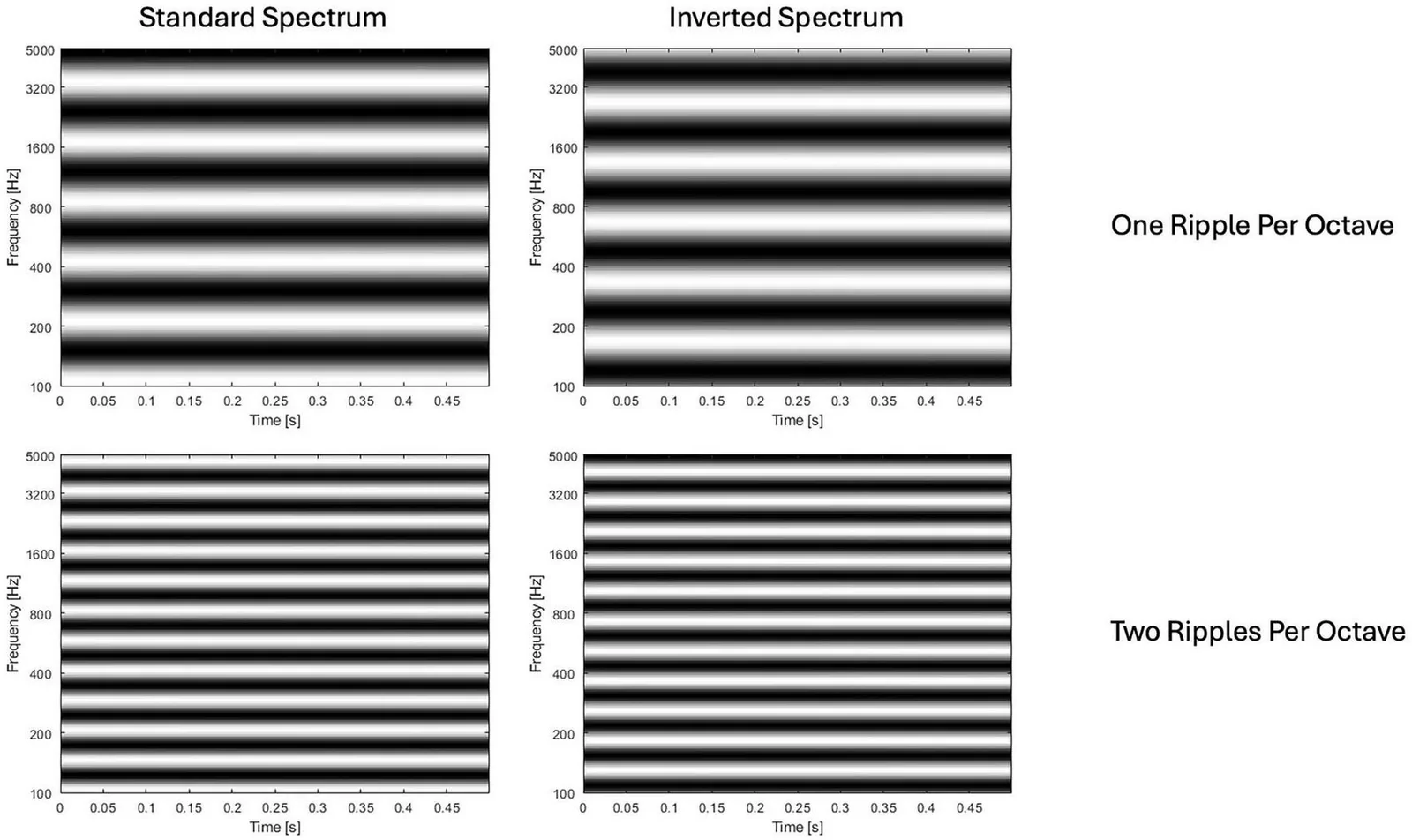
Spectrograms illustrating the spectral ripple discrimination (SRD) paradigm. Each panel shows a rippled stimulus in which spectral energy alternates between peaks and troughs across the frequency axis. The left column shows a standard ripple and the right column its phase-reversed counterpart, in which the positions of peaks and troughs are interchanged. The top row shows a low ripple density of one ripple per octave and the bottom row a higher density of two ripples per octave. The ripple pattern in each panel is fixed in frequency across the full stimulus duration, so that the SRD paradigm evaluates spectral modulation. The listener discriminates the difference between the standard stimulus and its phase-inverted version; the highest ripple density at which this difference is reliably detected defines the spectral ripple threshold, with higher thresholds reflecting finer spectral resolution.

One of the more extensively reported methods used to assess spectral resolution in CI users centers on the spectral ripple test, which employs ripple noise stimuli: broadband sound whose amplitude is varied sinusoidally along the frequency axis, so that the spectral envelope forms a regular alternation of peaks and troughs across the spectrum. The density of this variation is quantified in ripples per octave (RPO). A low-density stimulus presents a few broad peaks within each octave, whereas a high-density stimulus compresses many narrow peaks into the same frequency range, requiring finer frequency resolution to perceive. In SRD tasks, the listener detects the difference between a standard stimulus and a phase-inverted version in which the position of the peaks and troughs are interchanged. The maximum RPO at which a listener can reliably detect this phase reversal defines the spectral ripple threshold, with higher thresholds reflecting finer spectral resolution (Figure 4).

The SRD paradigm was introduced for use with CI populations by Henry and Turner (2003) and Henry et al. (2005) and quickly became the methodological standard: a 3-alternative forced choice (3AFC) task in which listeners identify the phase-inverted odd-one-out among three rippled noise stimuli. Won et al. (2007) provided the first clear evidence that this measure translated clinically, demonstrating significant correlations between spectral ripple thresholds and speech reception thresholds in both two-talker babble (*r* = −0.55) and steady-state noise (*r* = −0.62) in 31 CI users. Higher RPO thresholds, which reflected greater spectral resolution, predicted lower (i.e., better) speech reception thresholds. This was the first clear demonstration that a language agnostic measure of spectral discrimination ability correlates with speech recognition performance across multiple noise environments in CI users.

Anderson et al. (2011) extended this work in CI users, comparing ripple thresholds to spatial tuning curves, electrophysiological measures of electrode selectivity. Spatial tuning curves quantify the amount of electrical current (μA) required to mask a probe signal at a given electrode to provide a direct electrophysiological index of electrode interaction and channel selectivity within the implanted array.

The strong correspondence between the two (*r*^2^ = 0.79, *p* < 0.001, excluding two outliers) suggested that ripple discrimination and electrophysiologic measures capture related aspects of the same underlying limitation in spectral processing. Correlations with sentence recognition, however, were modest in quiet (*r* = 0.47–0.59) and absent in noise. This may reflect a stepwise dissociation as tests move away from controlled, precise measures to conditions that more closely resemble natural environments. Additionally, when nonlinguistic resolution measures are interpreted alongside speech testing, they can expose population-level differences in auditory processing, here between prelingually and postlingually deafened users, that speech scores alone leave invisible.

Understanding that CI users face constraints with spectral resolution in ways that HA users do not (e.g., limited electrode count, spread of excitation), Davies-Venn et al. (2015) examined whether spectral measures could similarly predict hearing and speech outcomes in NH and HI populations. Across NH and HI listeners, SMD thresholds at 2.0 cycles per octave correlated significantly with pure-tone average (*r* = 0.737, *p* < 0.001), unamplified word recognition scores (*r* = −0.498, *p* = 0.003), amplified syllable recognition (*r* = −0.741, *p* < 0.001), and QuickSIN (*r* = 0.596, *p* < 0.001). Notably, these correlations emerged at higher spectral densities than those typically observed in CI listeners, consistent with the relatively preserved frequency resolution available to HA users with mild to moderate HL compared to CI users. Building on prior work by Drennan et al. (2014) and Gifford et al. (2014) in the CI population, Davies-Venn et al. (2015) demonstrated that spectral modulation detection carries predictive value across a range of hearing levels and technologies, supporting its viability as a clinically informative tool across hearing populations.

#### 2.1.2 Spectral ripple discrimination in candidacy and cross linguistic application

Having established the spectral ripple paradigm and its relationship to speech recognition, two studies extend it beyond the postlingually deafened, English-speaking samples in which it was developed. The first asks whether SRD, administered unaided, can inform CI candidacy. The second asks whether its predictive value holds in a tonal language, where fine spectral structure carries greater phonemic weight than a non-tonal language (e.g., English).

Shim et al. (2014) examined SRD as a tool for CI candidacy assessment, testing the candidate ear under unaided listening conditions. SRD thresholds correlated strongly with aided AzBio Quiet sentence recognition (*r* = 0.77–0.80) and unaided W-22 word recognition (*r* = 0.74–0.76). Furthermore, SRD was used directly as a candidacy classifier against the gold standard of aided sentence recognition. They found that unaided thresholds predicted candidacy with an area under the ROC curve of 0.90 (95% CI ± 0.16), and in an independent eight-ear validation sample the candidacy decision derived from SRD aligned perfectly with the clinical determination. This demonstrated that an unaided, language-independent measure of spectral sensitivity may be able to meaningfully predict CI candidacy. Notably, SRD outperformed temporal measures in predicting speech perception and candidacy designation, a pattern reflecting the relative primacy of spectral cues over temporal cues in CI listeners. This assumption will be challenged when we compare auditory functions of prelingually versus postlingually deafened CI users later in this section and the Spectrotemporal section (cf. Gifford et al., 2018; Landsberger et al., 2019a).

The cross-linguistic reach of SRD was explored by Dai et al. (2018), who applied the ripple test to a Mandarin-speaking Chinese CI sample. Given its status as a tonal language, Mandarin presents particular demands on fine spectral structure. SRD thresholds correlated more strongly with Mandarin monosyllabic word recognition (*r* = 0.84, *p* < 0.001) than has generally been observed with English word recognition, while the correlation with sentence recognition was more consistent with prior literature (*r* = 0.54, *p* = 0.003). The authors suggest that spectral resolution may carry greater weight for speech recognition in tonal languages, where frequency distinctions are phonemically contrastive. This study extends the SRD’s validity to a non-Western, non-English-speaking population, an important extension of this work since sociolinguistic diversity and neurocognitive confounders are a global challenge. China’s linguistic diversity, for example, spans over 250 dialects and a large proportion of non-Mandarin speakers, mirroring the language access challenges faced in the United States, and makes this equity dimension directly relevant to the broader argument for language agnostic hearing assessment.

#### 2.1.3 Quick spectral modulation detection test

**FIGURE 5:**
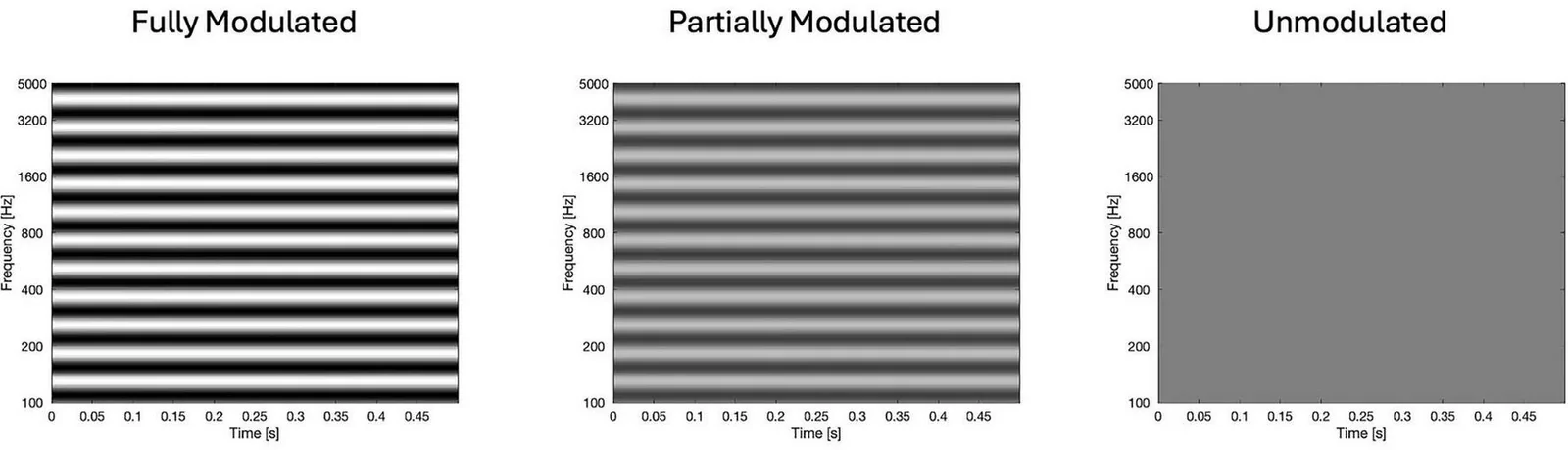
Spectrograms illustrating the spectral modulation detection (SMD) paradigm. Each panel shows a rippled spectrum whose modulation depth, the contrast between spectral peaks and troughs, is varied. The left panel shows a fully modulated stimulus with maximal peak-to-trough contrast, the center panel a partially modulated stimulus with reduced contrast, and the right panel an unmodulated stimulus with a flat spectral envelope. The smallest modulation depth that can be reliably detected defines the spectral modulation detection threshold, expressed in decibels.

Although both SRD and SMD use rippled noise stimuli, they differ in what is held constant and what is adaptively varied. SRD fixes modulation depth and tracks the highest ripple rate at which phase reversals remain discriminable, whereas SMD fixes ripple rate and tracks the minimum modulation depth at which spectral structure is detectable against an unmodulated noise reference (Anderson et al., 2012). The threshold is the smallest detectable modulation depth, expressed in decibels of peak-to-trough spectral contrast, with lower thresholds reflecting finer spectral resolution (Figure 5).

Building on earlier foundational work (Drennan et al., 2014; Henry and Turner, 2003; Won et al., 2011), Gifford et al. (2014) addressed a persistent barrier to clinical adoption of spectral modulation detection (SMD) testing by developing an abbreviated version, the Quick Spectral Modulation Detection (QSMD) test, that reduced administration time from 30 min to 6 while preserving psychoacoustic validity. QSMD scores correlated near-perfectly with full SMD scores (*r* = 0.90), demonstrating excellent test-retest reliability (*r* > 0.90). Furthermore, the QSMD predicted 66% of variance in CNC word recognition. Clinical accessibility was subsequently expanded with the EasyQSMD, a freely available software platform that produces equivalent outcomes using standard sound booth equipment (Landsberger et al., 2019b).

Kessler et al. (2020) tested the QSMD’s potential as a clinical tool, investigating whether SMD performance in the nonimplanted ear could predict bimodal speech benefit in CI users wearing a contralateral hearing aid. In 311 unilateral CI recipients recruited across three clinical sites, speech recognition was assessed using CNC monosyllables and AzBio sentences at +5 dB SNR under CI-alone and bimodal listening configurations. QSMD correlated with normalized acoustic benefit for CNC word recognition (*r* = 0.33, *p* < 0.0001) and AzBio sentence recognition at +5 dB SNR (*r* = 0.26, *p* = 0.002), such that listeners with better spectral resolution in the acoustic ear derived greater bimodal benefit. In a stepwise regression, however, QSMD, low-frequency pure-tone average (125–750 Hz), and age at implantation together accounted for only 19.1% of the variance in normalized speech benefit, and QSMD did not remain a significant independent predictor of sentence-in-noise outcomes after controlling for the remaining variables. These correlations were also less dramatic relative to earlier work on the topic by Zhang et al. (2013) which was also conducted in bimodal CI and hearing aid users (although this likely reflects the demographic heterogeneity of a large clinical population).

Thus far, the suggested clinical application is supplementary rather than substitutive. In patients who meet CI candidacy criteria bilaterally with comparable audiometric profiles, QSMD performance in the nonimplanted ear reflects residual spectral processing capacity not captured by pure-tone thresholds, and may serve as an additional input into the decision of which ear to implant and whether bimodal fitting is likely to confer benefit. Given that QSMD did not remain an independent predictor after accounting for audiometric and demographic factors, Kessler et al. (2020) is best read as establishing the feasibility of incorporating a language agnostic measure into bimodal fitting decisions rather than its adoption as a standalone criterion. These developments in spectral threshold tools such as the QSMD represent a meaningful step toward the practical integration of spectral assessment into routine CI care.

#### 2.1.4 Spectral resolution in prelingually deafened CI users

One caveat concerning spectral resolution testing applies to prelingually deafened CI populations. Gifford et al. (2018) administered the QSMD alongside CNC word recognition and AzBio sentence tests (in quiet and noise) to 578 CI users, including postlingually deafened adults, prelingually deafened adults, and prelingually deafened children. Although pediatric CI users demonstrated the highest average speech perception scores across all measures (outperforming postlingual adults by an average of 10, 15, and 30 percentage points on CNC words, AzBio in quiet, and AzBio in noise, respectively), they performed worst on the QSMD, scoring 15 percentage points below postlingual adults, with 21 of 36 children at or near chance and few exceeding 60% correct. Significant correlations between QSMD and speech measures were observed in both adult groups but not in pediatric CI users across any speech measure.

This raises the question: why would spectral resolution correlate with speech scores in prelingually deafened adults but not prelingually deafened children? The key differentiator appears to be early acoustic experience: all prelingually deafened adults in this sample wore hearing aids and communicated primarily through listening and spoken language prior to implantation, suggesting sufficient acoustic exposure to develop physiologically typical spectral processing pathways. The children in this sample, by contrast, underwent critical auditory development with the electrical input of a CI and appear to have organized their auditory systems around a different set of cues. A likely candidate is temporal envelope information, a possibility that the following section addresses.

Some caution is warranted in interpreting these findings. Spectral resolution does not fully mature in normal-hearing individuals until adolescence, and Davidson et al. (2021) demonstrated that speech perception scores in pediatric CI users continued to improve significantly between elementary and high school ages, suggesting that neural maturation may contribute to the poor QSMD performance independently of implant-specific factors. Floor effects in the pediatric QSMD data further limit the strength of the analysis. Taken together, these findings suggest that spectral resolution testing has constrained clinical utility in prelingually deafened children.

#### 2.1.5 Limitations of spectral resolution testing

A key interpretive limitation of spectral resolution testing concerns the phenomenon of spectral aliasing (also known as aliasing theory), the distortion of spectral information that occurs when the density of spectral content exceeds the resolving capacity of the implanted electrode array. Because CI processors rely on a limited number of electrodes, typically 12–22, the fine-grained frequency resolution that the intact cochlea achieves across thousands of hair cells is compressed into a coarser representation. When spectral content exceeds the resolving capacity of the electrode array, the output no longer faithfully reflects the intended spectral pattern. van Groesen et al. (2023) and Winn and O’Brien (2022) identified approximately 2–2.5 RPO as the practical ceiling of tonotopic fidelity in CI processors. Beyond this threshold, SMD scores may reflect non-spectral factors (e.g., temporal envelope tracking or task-specific learning) rather than spectral resolution alone. Restricting analyses to RPO values at or below this ceiling strengthens the correlation between spectral thresholds and speech outcomes, suggesting that linear correlations spanning the full RPO range can overestimate the clinical meaning of high-end scores. Importantly, the non-spectral cues that inflate spectral scores at higher ripple densities—particularly temporal envelope information—are themselves a distinct and clinically meaningful dimension of auditory function, one that the following section addresses directly.

### 2.2 Temporal sensitivity

Temporal processing is a fundamental dimension of auditory perception, distinct from but complementary to spectral resolution. Whereas spectral resolution concerns the ability to distinguish fine-grained differences in the frequency content of a sound, temporal resolution concerns the ability to detect and track changes in the amplitude of an acoustic signal over time. In speech, these temporal contours manifest as the rhythm of words and the prosody of a sentence as well as the voice onset time differences and formant transitions that distinguish individual phonemes.

Two primary paradigms have been used to quantify temporal sensitivity in CI populations. Amplitude modulation detection (AMD) measures the minimum change in amplitude, in decibels, that a listener can perceive at a fixed modulation frequency. Amplitude rate discrimination (ARD) measures the minimum change in modulation frequency, in hertz, that a listener can detect at a fixed modulation depth. Brochier et al. (2018) compared the two approaches in CI users and found that detection thresholds were highly correlated across participants (*r* = 0.92 at 10 Hz; *r* = 0.86 at 100 Hz), suggesting that both tasks rely on common temporal integration processes. Because AMD and ARD appear to index a common underlying capacity, temporal sensitivity in the following studies can be treated as a single construct in its relationship to speech outcomes, regardless of which paradigm a given study employed.

CI users are known to have significantly poorer temporal sensitivity than NH and HI listeners. The temporal modulation transfer function, which plots modulation detection sensitivity against modulation frequency, declines in all listeners as modulation frequency increases. Bacon and Viemeister (1985) showed that although NH and HI listeners perform comparably at low modulation frequencies, temporal sensitivity in the HI group declines more steeply than in the NH group as modulation frequency rises. This high-frequency disparity is more pronounced still in CI users, in whom the gap relative to NH and HI listeners emerges at lower modulation frequencies and widens more severely. In a cohort of 62 HA users, Kwak et al. (2018) showed that unaided temporal modulation detection thresholds correlated significantly with unaided speech discrimination scores in quiet (*r* = −0.324, *p* < 0.05) and unaided PTA (*r* = 0.303, *p* < 0.05). Temporal modulation detection thresholds did not differ between unaided and aided conditions, indicating that amplification did not improve temporal modulation sensitivity in this sample. The authors note that this pattern is consistent with a suprathreshold processing limitation that restoring audibility alone does not address although the present data do not localize the deficit. It may reflect degraded temporal encoding that amplification cannot repair, more central processing constraints, or both (Bernstein et al., 2016).

Won et al. (2011) provided the foundational evidence for acoustic temporal modulation detection as a valid measure of auditory function in CI users by demonstrating that temporal modulation detection thresholds (MDT) correlated significantly with both CNC word recognition (*r* = 0.65, *p* = 0.007) and speech reception thresholds in steady-state noise (*r* = 0.57, *p* = 0.03). Critically, MDTs showed no correlation with spectral ripple discrimination, suggesting that temporal and spectral sensitivity reflect fundamentally distinct auditory mechanisms. Furthermore, when combined in multiple regression, the two measures jointly accounted for 56% of the variance in CNC word recognition.

#### 2.2.1 Temporal modulation detection and neuroplasticity

Temporal resolution offers a useful window into auditory neuroplasticity, particularly the question of how the auditory system adapts to electrical hearing. In the wake of the findings by Gifford et al. (2018) that pediatric CI users perform better on speech tests despite worse spectral resolution than postlingually deafened adult CI users, Landsberger et al. (2019a) sought an explanation by comparing temporal modulation detection thresholds between pediatric and postlingually deafened adult CI users. The rationale was that adults who lose hearing after years of acoustic experience arrive at implantation with neural frameworks already shaped by natural sound, requiring reconfiguration to process electrical input. Children implanted early in development, by contrast, encounter electrical stimulation during the initial formation of auditory pathways, allowing those systems to organize around electrical input from the outset. Landsberger et al. (2019a) found that pediatric CI users demonstrated significantly better temporal modulation detection thresholds than postlingual adults, with mean thresholds of -13.9 dB versus -10.2 dB, respectively. This advantage was not correlated with age at implantation or duration of CI use within the pediatric group, suggesting it reflects a qualitative difference in auditory reorganization rather than simply more recent or extended implant experience. The finding aligns with the concept of cue reweighting: when detailed spectral information is unavailable, the auditory system may shift reliance toward temporal envelope cues, and when this shift occurs during early development, it appears to be more complete.

Taken together with the spectral findings from Gifford et al. (2018), these results illustrate how pediatric and adult CI users may arrive at similar speech recognition outcomes through distinct neurocognitive pathways, a fascinating distinction that language-dependent testing alone could not have revealed.

#### 2.2.2 Temporal sensitivity as a predictor of speech outcomes

Beyond characterizing group differences, a critical question for clinical practice is whether early temporal sensitivity can predict long-term speech recognition outcomes. Erb et al. (2019) addressed this by measuring amplitude modulation rate discrimination thresholds at 6 weeks post-activation and speech testing (in quiet only) at 6 weeks and 6 months post-activation. A modulation rate of 4 Hz was used as it falls within the frequency range most relevant to speech rhythm and prosody. Early modulation rate thresholds correlated significantly with speech recognition scores at 6 months (*r* = −0.51, *p* < 0.001), even after controlling for initial speech performance. Patients with poor early temporal sensitivity were approximately 17.6 times more likely to show poor speech recognition at 6 months.

Notably, early modulation rate discrimination was not correlated with speech scores obtained at the same time point. This dissociation likely reflects the different timelines of low-level temporal encoding and higher-order linguistic integration. Temporal sensitivity seems to emerge relatively early following implantation, while accurate word and sentence recognition requires additional time for auditory and linguistic systems to adapt and interact. In this respect, temporal sensitivity may offer a more accurate early signal of bottom-up auditory encoding integrity than conventional speech scores, which lack the specificity to isolate the individual elements that combine to form language perception.

For clinicians and patients alike, this has a practical implication: poor speech recognition scores in the early postoperative period do not necessarily portend poor long-term outcomes, particularly when early temporal sensitivity is intact. This perspective on early hearing performance may be useful for patient counseling and appropriate expectations setting during a period when patients and families are often most anxious about the trajectory of outcomes.

### 2.2.3 The EasyMDT

The research described above was conducted largely in laboratory settings, raising the practical question of how temporal sensitivity measures might be integrated into routine clinical assessment. Landsberger and Stupak (2022) addressed this by developing and validating the EasyMDT, a freely available software for the modulation detection threshold (MDT) test. In CI users, EasyMDT scores correlated strongly with CNC word recognition (*r* = −0.69), consonant identification (*r* = −0.82), and AzBio sentences in noise (*r* = −0.94). MDT performance did not correlate with vowel identification, consistent with the understanding that vowel perception in CI users draws more heavily on spectral cues than on temporal envelope information. Equally notable was the absence of a correlation between MDT and scores on the Spectral Modulation Ripple Test (SMRT)—-a spectrotemporal test that is covered in the next section—-confirming that temporal and spectral sensitivity reflect distinct auditory mechanisms and provide non-redundant information about CI function. Together, these findings position the EasyMDT as a practical and informative tool for comprehensive hearing assessment in CI candidates and users.

However, the clinical generalizability of the temporal sensitivity is nevertheless constrained by a significant gap: validation across hearing device configurations and HI populations. The three primary studies underlying its validation in CI populations enrolled CI-only samples (Gifford et al., 2018; Landsberger et al., 2019a; Landsberger and Stupak, 2022). In all three studies, participants with residual hearing in the non-test ear were assessed without their hearing aids and/or with occlusion devices. Gifford et al. (2018) justified this approach on the basis of standardized unilateral testing conditions, ensuring that bilateral CI and bimodal users could be evaluated on a consistent basis. Future research conducted with multiple hearing device configurations (e.g., unilateral CIs, bilateral CIs, and CI+HA), reflecting the methodology chosen by Lawler et al. (2017), would help shape our understanding of temporal resolution testing across the spectrum of HI populations.

Across these studies, temporal sensitivity emerges as a distinct dimension of the language agnostic framework rather than a redundant proxy for spectral resolution. Because it can be assessed early, in some cases before speech measures stabilize, and probes the integrity of envelope encoding independent of linguistic knowledge, temporal sensitivity offers a complement to the spectral measures examined above and the existing clinical battery. Its contribution to the broader objective is twofold: it can potentially act as a language-independent signal of postoperative trajectory, and it is the domain in which developmental differences between prelingually and postlingually deafened listeners become most apparent, a distinction speech scores alone can obscure.

### 2.3 Spectrotemporal resolution

**FIGURE 6:**
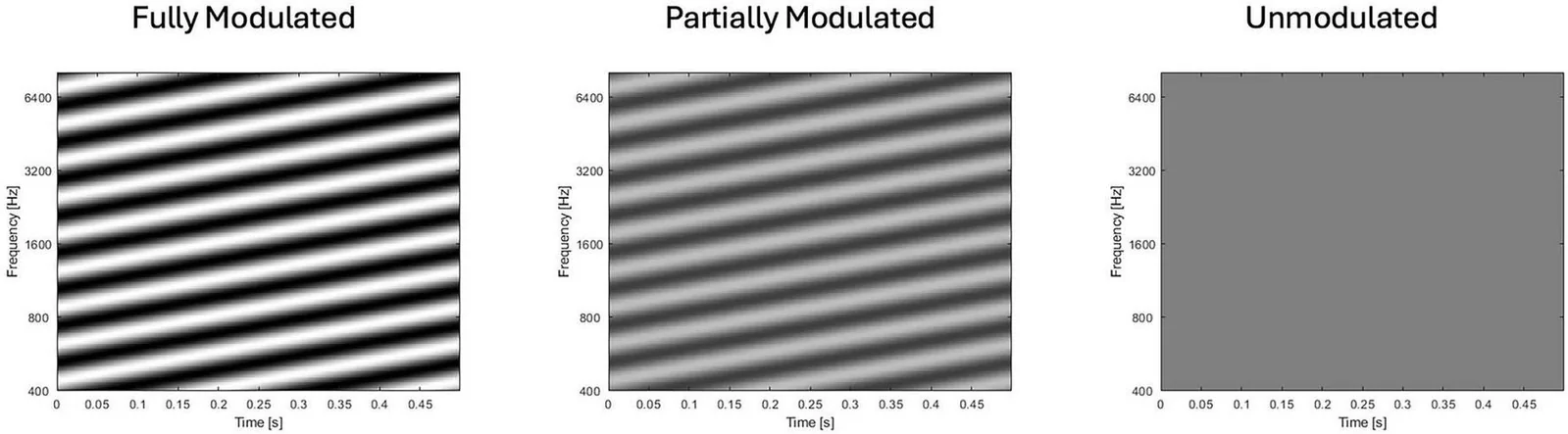
Spectrograms illustrating a spectrotemporal modulation (STM) detection paradigm. Each panel shows a rippled spectrum that drifts across frequency over time, at a temporal modulation rate of 4 Hz and a spectral density of 2 ripples per octave (RPO), while its modulation depth, the contrast between spectral peaks and troughs, is varied. The left panel shows a fully modulated stimulus with maximal peak-to-trough contrast, the center panel a partially modulated stimulus with reduced contrast, and the right panel an unmodulated stimulus with a flat spectral envelope. These stimuli exhibit movement of the ripple pattern in time as well as in frequency. The smallest modulation depth that can be reliably detected defines the STM detection threshold, expressed in decibels.

The preceding sections examined spectral and temporal processing as distinct dimensions of auditory function. In practice, however, these two cues interact to create spectrotemporal sensitivity, the ability to detect and resolve modulations that vary simultaneously across both frequency and time. This is a measure that more closely approximates the dual demands of everyday listening. Several tests have been developed to assess this capacity in CI users, each designed to probe the auditory system’s ability to track dynamic changes in the spectral envelope over time. Figure 6 depicts one type of spectrotemporal modulation stimulus in which a rippled spectrum drifts across frequency over time while its modulation depth is varied to establish a detection threshold. The smallest modulation depth that can be reliably detected defines the spectrotemporal modulation detection threshold, expressed in decibels.

#### 2.3.1 The Spectrotemporally Modulated Ripple Test (SMRT)

The Spectrotemporally Modulated Ripple Test (SMRT) was developed by Aronoff and Landsberger (2013) as a methodological improvement over existing static spectral ripple paradigms. In the static version of the test where listeners are asked to discriminate between two phase-inverted stimuli, a task can, in principle, be accomplished through non-spectral cues (e.g., loudness, spectral centroid, or edge-intensity cues). The SMRT addresses this limitation by introducing a continuously drifting phase, which converts the static spectral modulation pattern into a steady-state amplitude modulation. This design makes the task more specifically dependent on the listener’s ability to track changes in spectral shape over time. Results are expressed as ripples per octave (RPO), in which higher thresholds reflect finer spectrotemporal resolution.

Initial validation of the SMRT in CI users demonstrated strong relationships with standard clinical speech measures. Lawler et al. (2017) compared SMRT scores to AzBio (+8 dB SNR) sentence recognition in a sample of 25 CI users (eight using unilateral CIs, nine using bilateral CIs, and eight using a CI and a hearing aid). The mean SMRT threshold was 3.136 RPO (95% CI: 2.74–3.54), with a range of 0.5–8.3 RPO, and they found a significant correlation between SMRT and sentence scores (*r* = 0.83, 95% CI = 0.63–0.94), with each additional RPO corresponding to approximately a 12% increase in AzBio (+8 dB SNR) performance. No significant difference in the SMRT-AzBio relationship was found pairwise between any of the three groups. This finding is further supported by comparing findings by Lawler et al. (2017) to Holden et al. (2016) who investigated the SMRT-AzBio relationship in unilateral CI conditions only. The slopes and intercepts of the correlations are remarkably similar between groups and studies, suggesting that the relationship between SMRT and AzBio remains consistent across different CI populations. As suggested by Lawler et al. (2017), such an observation would benefit from further research across different HI populations. The study also described a phenomenon termed “temporal smearing,” in which increasing channel overlap at higher ripple densities diminishes the within-channel amplitude modulation that the implant can faithfully encode. In this case, the stimulus changes faster than any individual electrode channel can track, progressively degrading the perceptual signal, resulting in an asymptotic performance below that of normal-hearing listeners.

Cross-linguistic applications of the SMRT have provided additional support for its generalizability as a language agnostic assessment tool. Jeddi et al. (2019) administered the SMRT alongside a frequency discrimination threshold (FDT) test and Persian-language word and sentence recognition measures in a sample of 75 pediatric CI users. The SMRT demonstrated stronger associations with both word recognition (*r* = 0.573, *p* < 0.001) and sentence perception (*r* = 0.556, *p* < 0.001) than the FDT alone. Together, the SMRT and FDT accounted for approximately 41% of variance in word recognition and sentence perception, independently. That spectrotemporal resolution should explain a meaningful share of speech performance even in non-Western, pediatric, and prelingually deafened populations reinforces the clinical relevance of these measures.

In another non-English speaking CI population, van Groesen et al. (2023) compared the SMRT, STRIPES (described in the following section), and a matrix speech-in-noise test in a Dutch and Flemish speaking CI population. They found that both measures accounted for a modest but statistically significant 28% of the variance in speech-in-noise recognition thresholds (*p* = 0.04). The authors concluded that both tests serve as valid and efficient indicators of across-listener differences in CI performance, even as the modest effect sizes underscore that spectrotemporal resolution is one of several contributing factors that shape listening performance in noise.

#### 2.3.2 Spectrotemporal Ripple for Investigating Processor EffectivenesS (STRIPES)

Whereas the SMRT holds ripple density fixed and drifts the ripple phase over time, the Spectrotemporal Ripple for Investigating Processor EffectivenesS (STRIPES) requires listeners to discriminate sounds that sweep upward versus downward in frequency, with task difficulty titrated by varying the spectrotemporal density of the sweeps. Because the test is constructed to minimize extraneous cues so that performance depends only on resolving sweep direction, STRIPES was designed to be a direct probe of integrated spectrotemporal processing and of how effectively a given processor conveys it.

STRIPES was developed and validated by Archer-Boyd et al. (2018) in a small sample of normal-hearing and CI listeners, with subsequent validation in clinical settings reported by Archer-Boyd et al. (2020). A third study by Archer-Boyd et al. (2023) extended this validation to telehealth administration, finding no significant differences in performance between remote and in-clinic conditions and offering a practical finding to support the potential for spectrotemporal testing in the context of telehealth or hybrid audiology care models. In a small sample of CI users (*n* = 7), Bysouth-Young et al. (2025) compared STRIPES to the British Coordinate Response Measure (CRM), a validated speech-in-noise test used for occupational auditory fitness-for-duty assessment, finding no significant relationship between the two measures, though the small sample limits the interpretive weight of this finding.

Taken together, STRIPES has been validated across laboratory, clinical, and telehealth settings, supporting its feasibility as a deployable spectrotemporal measure. Its relationship to speech outcomes, however, remains less established than that of the SMRT. The absence of a significant association with the CRM, though limited by a sample of seven, underscores that its predictive validity for everyday listening has not yet been demonstrated as convincingly as its psychometric robustness.

#### 2.3.3 Spectrotemporal modulation detection

The majority of studies reviewed in this section assess spectrotemporal processing as a predictor of outcomes in already-implanted CI recipients. Choi et al. (2016) occupies a distinct position by targeting the pre-implantation phase, asking whether an unaided, non-linguistic spectrotemporal modulation (STM) detection test could serve as a surrogate for the conventional aided speech recognition battery used to establish CI candidacy. In a preliminary feasibility study, the authors enrolled 27 Korean-speaking adults with moderately severe to profound bilateral sensorineural hearing loss and administered STM detection thresholds across six stimulus conditions varying in spectral density and temporal modulation rate. Notably, STM testing was conducted without hearing aids while speech recognition was assessed using the Korean Central Institute for the Deaf sentence test (K-CID) under aided listening conditions. The approach was motivated by the clinical burden of establishing best-aided listening conditions and justified by the methodological precedent of Shim et al. (2014), who demonstrated that unaided spectral ripple discrimination correlated significantly with aided speech recognition in listeners with hearing impairment. Choi et al. (2016) replicated this correlational finding as an independent confirmation of the finding of Shim et al.

They found that STM detection thresholds at 0.5 cycles per octave (c/o) and 5 Hz showed the strongest correlation with K-CID sentence scores (*r* = −0.762, *p* < 0.0001). This stimulus condition seemed to approximate the acoustic characteristics of conversational speech, as temporal modulation rates in the 4–8 Hz range correspond to syllabic rhythm, while spectral densities near 0.5 c/o align with the coarse formant structure that distinguishes vowels. Using a K-CID score of ≤ 50% as the CI candidacy criterion, ROC analysis yielded an area under curve of 0.83 (sensitivity = 78.6%, specificity = 76.9%, accuracy = 77.8%), indicating reasonable discriminability between candidates and non-candidates. When candidacy classification was based on all six STM detection thresholds, only three of the 27 listeners were classified discordantly from the clinical decision, however these three listeners each retained relatively preserved hearing in one ear. Interpretation of these estimates warrants caution: the sample of 27 participants limits the stability of the ROC parameters, and the ≤ 50% K-CID threshold, while clinically plausible, was selected for this study rather than derived from candidacy guidelines. These constraints position the findings as exploratory rather than confirmatory. Nonetheless, the results suggest that an unaided, language agnostic psychoacoustic measure may approximate the results of a more resource-intensive candidacy evaluation without requiring aided conditions or language-matched speech materials. Rather than replacing the standard battery outright, the STM detection test may be better positioned as a viable first-pass screening tool, particularly in settings where speech measures are confounded by resource constraints, cognitive ability, educational background, or limited linguistic familiarity with the test materials, pending replication in larger and more demographically diverse samples.

#### 2.3.4 The audible contrast threshold test

One such test has found its role as a clinically viable, commercially available spectrotemporal measure: the Audible Contrast Threshold (ACT). The ACT operationalizes Plomp’s two-component model of hearing loss, in which a distortion component, independent of audibility, accounts for residual speech-in-noise difficulty that amplification alone cannot resolve (Bernstein et al., 2013; Plomp, 1986; Sanchez-Lopez et al., 2020). Building on prior demonstrations that STM thresholds correlate with speech-in-noise reception in listeners with hearing loss (Bernstein et al., 2016; Davies-Venn et al., 2015), Zaar et al. (2023) developed a modified STM paradigm with elderly listeners in mind. Special attention was paid with this population in mind because they (a) account for the greatest proportion of hearing aid users; (b) are positioned, from a neurocognitive standpoint, to be especially benefited by LAHTs; and (c) earlier STM procedures had suffered high non-completion rates in this group (Bernstein et al., 2013, 2016; Elgaddal and Weeks, 2023). Zaar et al. (2024a) subsequently established that STM thresholds accounted for 61% of aided SRT variance (*R*^2^ = 0.61), exceeding the predictive value of the 4-frequency PTA (*R*^2^ = 0.51), with complementary predictions in a combined model (*R*^2^ = 0.69). STM thresholds also accounted for 51% of the variance in the SRT benefit conferred by directional microphone and noise reduction processing (*R*^2^ = 0.51, *p* < 0.001), identifying the measure as a predictor of both baseline speech reception and the degree to which a patient stands to benefit from advanced hearing-aid signal processing.

These findings enabled the ACT’s clinical formalization (Zaar et al., 2024b). The ACT replaced earlier 3-AFC STM formats with a simpler detection task: the patient presses a pushbutton upon hearing a modulated target embedded in continuous, audibility-compensated noise, tracked via a Hughson-Westlake procedure analogous to standard pure-tone audiometry. The test is transducer-agnostic, requires no equipment beyond a standard audiometric setup, and is language agnostic by design. Mean test duration is approximately 75 s. Results are expressed on the normalized Contrast Level scale (dB nCL), where 0 ± 4 dB nCL represents normal contrast sensitivity; values above 4 dB nCL indicate progressive contrast loss up to a ceiling of 16 dB nCL.

This test is used to guide hearing-aid fitting, with higher contrast loss indicating greater expected benefit from directional and noise-reduction processing. Because the ACT and the audiogram evaluate distortion and audibility, respectively, they constitute complementary, language-agnostic measures of hearing function, both administrable within a standard clinical encounter. The ACT is currently integrated into three Interacoustics audiometer platforms (Affinity Compact, Callisto, Equinox Evo) and is in clinical use in Europe (Interacoustics 2023). Its clinical case rests on predictive validity: STM thresholds account for a majority of the variance in aided speech reception and predict the benefit conferred by advanced signal processing (Zaar et al., 2023, 2024a). The primary limitation of the ACT is the absence of prospective evidence that ACT-guided fitting yields better patient-reported outcomes or speech recognition than standard audiogram-based fitting. However, research on the ACT continues to evolve, with recent work evaluating user-operated, automated implementations intended to support self-administered testing in clinical settings (Sanchez-Lopez et al., 2026). Work is also underway to extend the ACT beyond behavioral administration as an electrophysiological version based on auditory change complex and is considered among the electrophysiological measures below.

#### 2.3.5 Pediatric considerations in spectrotemporal testing

The relationship between spectrotemporal processing and hearing outcomes in children with CIs differs meaningfully from patterns observed in adults. Landsberger et al. (2018) compared SMRT performance across children and adults with CI and normal hearing (NH), finding that adults with CI consistently outperformed children with CI (4.3 vs. 3.06 RPO), while NH children reached adult-like performance by age 10. CI children, by contrast, showed no meaningful relationship between age, duration of CI use, and SMRT performance, suggesting that the coarser spectral coding resulting from electrical stimulation from the onset may noticeably constrain the maturation of spectral processing.

Taken together, Gifford et al. (2018) and Landsberger et al. (2018) established that children with CIs underperform on spectral and spectrotemporal threshold tests despite achieving speech recognition scores comparable to adult CI users. Landsberger et al. (2019a) offered a partial explanation, demonstrating that pediatric CI users exhibit significantly better temporal modulation detection than adults, suggesting a cue re-weighting in which children compensate for reduced spectral resolution by drawing more heavily on temporal envelope information. DeFreese et al. (2024) extended this line of inquiry, finding no significant correlation between spectral or temporal resolution and speech recognition across a broad battery of measures (CNC quiet, BabyBio +5 and 0 SNR, BKB-SIN, HINT, and vowel recognition). These findings converge on the view that children with prelingual deafness appear to develop novel strategies for speech processing, ones that are not well defined by either standard speech measures or the non-linguistic tasks that reliably predict hearing performance in adults. The authors note that the moderate effect sizes for temporal sensitivity on vowel and speech-in-noise tasks in this sample of 47 children may become statistically meaningful in larger or longitudinal samples (see Table 2). Nonetheless, these findings reflect meaningful progress. By pairing psychoacoustic resolution measures with standard speech testing, researchers have been able to further characterize the unique perceptual architecture of pediatric CI hearing in ways that speech scores alone have not.

**TABLE 2:** Reprinted from “The impact of spectral and temporal processing on speech recognition in “*children with cochlear implants*,” by DeFreese et al. (2024), *Scientific Reports*, *14*, 14094 [Table 1: “Correlation coefficients (R) from partial correlations (controlling for duration of daily processor use).

	Speech recognition test						
Modulation detection type	Test condition	CNC (RAU)	BabyBio +5 dB (RAU)	BabyBio 0 dB (RAU)	BKB-SIN (dB SNR-50)	HINT RSPACE (dB SNR)	Vowels
Spectral	0.5 cyc/oct	– 0.131 (*N* = 40)	– 0.348 (*N* = 40)	– 0.243 (*N* = 35)	0.174 (*N* = 40)	0.074 (*N* = 40)	– 0.427 (*N* = 24)
	1.0 cyc/oct	– 0.003 (*N* = 18)	– 0.421 (*N* = 8)	– 0.333 (*N* = 18)	0.120 (*N* = 18)	0.015 (*N* = 18)	– 0.445 (*N* = 18)
Temporal	4 Hz	0.102 (*N* = 42)	– 0.214 (*N* = 42)	0.017 (*N* = 36)	– 0.030 (*N* = 42)	0.029 (*N* = 40)	– 0.439 (*N* = 24)
	32 Hz	– 0.125 (*N* = 18)	– 0.332 (*N* = 18)	– 0.300 (*N* = 18)	0.263 (*N* = 18)	0.303 (*N* = 18)	– 0.337 (*N* = 18)
	128 Hz	– 0.011 (*N* = 18)	– 0.175 (*N* = 18)	– 0.018 (*N* = 18)	0.049 (*N* = 18)	0.115 (*N* = 18)	– 0.052 (*N* = 18)

Following correction for multiple comparisons, none of the correlational analyses reached statistical significance.”]. The formatting of the original table has been revised to include column headers for ‘Modulation Detection Type’ and ‘Test Condition,’ in place of the original merged row labels. Copyright 2024 by the Authors. Licensed under CC BY 4.0 (https://creativecommons.org/licenses/by/4.0/). RAU, rationalized arcsine units.

#### 2.3.6 Psychoacoustic resolution tests and hearing aid users

The preceding measures were developed and validated predominantly in CI users, in whom spectral, temporal, and spectrotemporal resolution thresholds consistently predict speech recognition. A smaller body of work extends these paradigms to HA users and the broader HI population (Davies-Venn et al., 2015; Kwak et al., 2018). In HA and HI listeners, the same testing modalities used to measure resolution thresholds of devices constrained by limited electrode count and spread of excitation are adapted to probe the suprathreshold distortion that amplification does not resolve. The clinical maturity of these measures has diverged significantly: the spectrotemporal modulation paradigm has reached routine clinical use only in the hearing aid context, where the Audible Contrast Threshold can now guide HA fitting, whereas the CI-oriented resolution measures remain largely research tools (Zaar et al., 2024b).

### 2.4 Overall limitations and interpretive considerations for psychoacoustic resolution tests

The preceding sections evaluated spectral, temporal, and spectrotemporal measures individually. Two interpretive cautions, however, warrant consideration across all stimulus types and bear on how any psychoacoustic resolution threshold should be read. The first concerns the fidelity with which CI processors reproduce the stimuli themselves; the second concerns whether spectrotemporal tasks measure the integrated faculty they are presumed to capture. Both of these considerations temper the reported correlations and shape how these measures may be positioned within a clinical battery.

#### 2.4.1 Aliasing theory

As discussed in the limitations of spectral resolution testing section, CI signal processing cannot faithfully represent spectral modulation above approximately 2–2.5 RPO, becoming non-monotonic and limiting the interpretive validity of thresholds. In an analysis of 34 published studies, Winn and O’Brien (2022) found that 73% of reported SMRT thresholds fell above this aliasing limit, raising questions about what is being measured in that performance range. Re-analysis of existing datasets showed that restricting scores to the sub-aliasing range ( ≤ 2 RPO) strengthened correlations with speech recognition outcomes.

#### 2.4.2 Spectral vs. temporal contributions to SMRT performance

Whether spectrotemporal ripple measures capture a genuinely integrated spectrotemporal faculty or reflect primarily spectral or temporal processing remains an open question. Landsberger and Stupak (2022) found no significant correlation between MDT performance and SMRT scores in CI users, interpreting this as evidence that the two tasks engage distinct cue types processed by quasi-independent neurocognitive pathways. Spectrotemporal ripple discrimination may depend more on spectral resolution than temporal sensitivity.

Zhou et al. (2020) reported a contrasting pattern. In their CI sample, SMRT performance was strongly correlated with temporal sensitivity (*r* = −0.68, *p* = 0.002), accounting for 43% of the variance, whereas SMRT was not significantly correlated with a ripple discrimination task (*r* = 0.03, *p* = 0.93). After statistically controlling for amplitude modulation sensitivity, the relationship between SMRT performance and speech recognition disappeared; controlling for ripple discrimination left it intact. The authors interpreted this as evidence that SMRT thresholds are driven primarily by temporal modulation cues rather than spectral resolution, a conclusion that would substantially complicate the interpretation of SMRT as a spectrotemporal measure.

These findings are not easily reconciled and may reflect differences in participant characteristics, stimulus parameters, or the specific modulation rates employed. Together, they underscore that the validity of spectrotemporal ripple measures remains an open question, with meaningful consequences for how results are interpreted clinically and how these measures are positioned within a broader language agnostic assessment battery.

## 3 Naturalistic tests

Whereas psychoacoustic measures examine hearing with controlled stimulus modulations, the measures in this section employ more ecologically similar stimuli: environmental sounds, music, and the phonemes and logatomes that constitute the building blocks of speech. These stimuli trade some of the mechanistic precision of ripple and modulation tasks for ecological validity. Within the aims of this review, they extend the language agnostic framework beyond peripheral encoding to examine more functional consequences of hearing loss.

### 3.1 Familiar environmental sounds test—identification

Beyond the perception of speech, the ability to identify familiar sounds in the environment (e.g., dog barking, siren approaching, water running) represents a fundamental dimension of auditory function with direct implications for safety and independence. The Familiar Environmental Sound Test–Identification (FEST-I) was developed to provide a standardized, closed-set measure of this capacity. The test originated from a 192-item pool representing 48 sound sources, validated in normal-hearing listeners at 95% accuracy (Shafiro, 2008a). Vocoder simulations demonstrated that environmental sound identification improved with increasing spectral resolution up to approximately eight frequency channels but plateaued beyond that point a ceiling with particular relevance for CI users because the number of frequency channels in modern CI processors falls above that range (Shafiro, 2008b).

When the FEST-I was administered to experienced CI users, performance was markedly reduced relative to normal-hearing peers. Shafiro et al. (2011) reported a mean accuracy of 45.3% (SD = 16.2) across 17 postlingually deafened adults, compared with > 95% accuracy in controls. Harris et al. (2017) found a mean of 61% accuracy (SD = 14.96) in 35 experienced CI users against 90% (SD = 5.22) for age-matched controls, with one out of six CI users performing within the normal-hearing range. FEST-I scores correlated strongly with speech recognition, including Perceptually Robust English Sentence Test Open-set (PRESTO) sentences (*r* = 0.795, *p* < 0.001) and CID-W22 words (*r* = 0.762, *p* < 0.001), both in quiet conditions. These findings suggest that environmental sound awareness and speech perception draw on shared auditory and cognitive processes.

Whether environmental sound identification improves following implantation remains an open question. McMahon et al. (2018) compared 39 experienced CI users with 20 CI candidates and found no significant difference in accuracy (59.9% vs. 54.7%), suggesting limited benefit from device experience alone. The real-world stakes of this deficit are particularly salient when isolating safety-relevant sounds from the FEST-I (e.g., fire alarm, ambulance, thunder), and CI users and candidates correctly identified 57–68% of sounds compared to 95.1% for normal-hearing controls (Hamel et al., 2020; Luzum et al., 2023). A systematic review synthesized 19 studies and confirmed these patterns broadly, reporting accuracy ranging from 31 to 87%, wide individual variability, and no systematic evidence of improvement following implantation (Shafiro et al., 2022). These deficits are not merely functional, as severe and profound hearing loss has been associated with elevated all-cause and trauma-related mortality, underscoring the importance of accurate environmental sound identification (Kim et al., 2020; Choi et al., 2024).

For the clinician, these findings underscore that environmental sound perception represents a dimension of auditory function that standard speech batteries may not capture yet that matters for patient safety. Observed bimodal advantages and correlations between FEST-I and speech perception scores suggest that environmental sound testing may serve as a complementary, language agnostic metric for characterizing overall auditory capacity and optimizing patient safety.

### 3.2 Logatome and phoneme discrimination

Whereas psychoacoustic measures of spectrotemporal resolution and environmental noise identification represent non-speech acoustic stimuli that do not resemble speech, logatomes and phonemes are language-adjacent voice productions that can be used to evaluate hearing performance without the confounds of linguistic familiarity. Phonemes are the smallest unit of sound in a language that can distinguish meaning between words (e.g., /b/ and /k/ in “bat” and “cat”) while logatomes are nonsense pseudowords (e.g., “snarp,” “ostail”) (Welge-Lüßen et al., 1997). These stimuli occupy a middle ground between purely non-linguistic sounds and conventional speech testing.

Rahne et al. (2010) developed a logatome discrimination test using the Oldenburg Logatome Corpus (OLLO), a multilingual database of consonant-vowel-consonant (CVC) or vowel-consonant-vowel (VCV) recordings from 40 German speakers. The test presented 100 logatome pairs in a same-different, forced-choice paradigm. The authors administered the test alongside the Oldenburg Sentence Test (OLSA) to 13 normal-hearing adults and 8 CI users. All participants were able to complete the logatome discrimination task, whereas only the four highest-performing CI users could complete the OLSA due to a floor effect of the sentence test. Among CI users, performance in the logatome task varied from chance level to near-perfect performance, providing substantially greater resolution across the performance range than the sentence test.

Ching et al. (2021) developed the Language Independent Test of Auditory Discrimination (LIT-AD), a phoneme-based test designed for CI candidacy referral. The test used VCV stimuli spoken by a female speaker, presented in a 3AFC task format in which each triplet contained two productions of the same VCV syllable and one differing in either the vowel or consonant. Each phoneme contrast was individually adjusted in signal-to-noise ratio so that the average CI user achieved approximately 70% correct. In the evaluation of 81 adults (41 HA users, 40 CI users), LIT-AD scores correlated significantly with sentence-in-noise tests for both HA users (*r* = −0.54, *p* < 0.001) and CI users (*r* = −0.73, *p* < 0.001). An interesting dissociation emerged among HA users: some who scored below average on phoneme discrimination still achieved above-average sentence scores, likely reflecting the contribution of top-down linguistic cues that are absent in the phoneme discrimination task. This pattern illustrates one kind of advantage a language-independent measure provides: by isolating access to speech cues that are normally present in speech, the LIT-AD more closely targets the auditory component of speech perception. Using a regression model adjusted for duration of hearing loss, Ching et al. (2021) estimated the probability that a given HA user would achieve superior LIT-AD performance with a CI, finding, for example, that a score of 60% correct while wearing HAs corresponded to a 93% probability of improved discrimination with a CI. Notable limitations of this study include (a) limited generalizability beyond postlingually deafened adults; (b) a small dataset to generate a curve reliably predictive of CI benefit; and (c) although the stimuli were designed to include cross-phonemic consonants, the test was evaluated with English speaking adults only in a lab setting. Additionally, the authors found that some HA users scored below average on the LIT-AD but above average on sentence perception. The authors note that switching from HA to CI could improve consonant discrimination while potentially *worsening* access to prosodic cues, meaning the LIT-AD may overestimate functional sentence-level benefit in some patients.

### 3.3 Music

Music perception and enjoyment is another lens through which CI outcomes can be understood as language agnostic. Although lyric-bearing music engages listeners through linguistically encoded content, much of music is instrumental, and even lyric-containing songs are experienced substantially through melody, rhythm, timbre, and harmonic structure that operate independently of language. To the extent that music enjoyment relies on these non-linguistic features, music perception tasks qualify as language agnostic outcome measures. Music enjoyment also offers a notable window into the experience of CI users: about one-third of CI users report stopping listening to music altogether following implantation, and deficits in music perception and enjoyment are among the most consistently documented quality-of-life consequences of cochlear implantation (Looi et al., 2012; Mirza et al., 2003).

The view that music is sociolinguistically neutral, however, requires qualification. Even instrumental music carries cultural assumptions: familiarity with tonal conventions, genre exposure, and prior musical experience all influence performance on music perception tasks, complicating efforts to isolate peripheral auditory function from neurocognitive confounders (learned musical knowledge, for example). These constraints may not disqualify music perception from LAHT consideration entirely, but they highlight the need for careful stimulus development and cross-cultural validation before music-based assessments can be applied equitably across diverse patient populations (Hwa et al., 2021).

At the psychoacoustic level, CI users face well-documented challenges with the acoustic features central to music perception. Pitch and timbre resolution are severely limited by the reduced number of active electrodes and their relatively broad spread of excitation (“current spreading”). The normal cochlea supports approximately 1,400 discriminable pitch steps across the audible range (Zeng et al., 2014); by contrast, current-steering strategies such as the Advanced Bionics HiRes Fidelity 120 produce up to 120 spectral channels, representing a greater than 10-fold reduction in spectral resolution at the processor level (Koch et al., 2007). Channel counts vary by manufacturer and model, and this measure does not correspond reliably to the listener’s effective spectral resolution. However, this gap between acoustic and electrical stimulation cannot be closed simply by increasing electrode or virtual channel count alone, a finding substantiated by, among other measures, the use of spectral modulation detection thresholds (Berg et al., 2019, 2020; Friesen et al., 2001; Gifford et al., 2018).

The perceptual consequences of this limitation are evident in objective music perception tasks. In the Clinical Appreciation of Music Perception (CAMP) test, CI users achieved melody identification accuracy of only 15.7–48% and timbre accuracy of 25.5–63.7%, compared to 72 and 87%, respectively for normal-hearing listeners (Nimmons et al., 2008). Importantly, objective perception scores do not always align with subjective enjoyment: some CI users report meaningful musical pleasure despite poor discrimination performance, suggesting that enjoyment employs cognitive and emotional processes beyond peripheral auditory resolution. Patient-reported outcome measures targeting music enjoyment therefore capture a dimension of CI benefit that objective perception tasks alone cannot. This disconnect between objective measure and quality of life is a common thread when evaluating hearing function in the CI population.

Furthermore, the field lacks a standardized assessment tool. Hwa et al. (2021) identified twelve validated instruments spanning objective and subjective domains, yet none has achieved widespread adoption. Music perception and enjoyment offers a considerably language agnostic window into CI outcomes, though its clinical utility remains constrained by the absence of a standardized assessment tool and limited systematic investigation.

### 3.4 Naturalistic testing on hearing aid listeners

The naturalistic measures reviewed here focused mainly on CI users with normal-hearing controls, and they remain largely underinvestigated in the HA population. Environmental sound identification and music perception have been studied in CI recipients and CI candidates with NH listeners as controls, but with sparse comparable HA data. Phoneme discrimination is one naturalistic measure with HA data, but that sample was tested chiefly to inform cochlear implant candidacy referral rather than to characterize hearing aid outcomes (Ching et al., 2021). Validating these measures in hearing aid and hearing-impaired listeners is an important direction for future research.

## 4 Objective electrophysiologic measures of hearing

Unlike the behavioral measures examined above, the measures in this section are unified not by the presented stimuli but by the method of response assessment. These measures record the auditory system’s neural response directly, from the brainstem to the cortex, and require no behavioral reporting. This property makes them especially relevant for prelingually deaf pediatric CI users, populations who cannot reliably engage in behavioral tasks, and as research tools. They measure neural responses rather than perceptual response and therefore provide a coarser readout than the graded thresholds of behavioral testing. Nonetheless, these tests serve our aim of characterizing auditory function independent of linguistic and cognitive demand, and they occupy a distinct and potentially valuable category of tools within the LAHT taxonomy.

At the subcortical level, the auditory brainstem response (ABR) measures the integrity of the auditory nerve and brainstem and serves as a primary screening tool in combination with otoacoustic emissions (OAEs) in newborn screening (Joint Committee on Infant Hearing, 2019). In CI users, the electrical auditory brainstem response (EABR) adapts this paradigm by delivering the stimulus directly through a CI electrode, enabling intra- and post-operative monitoring of neural integrity (Brown et al., 1994). The auditory steady-state response (ASSR) is a distinct electrophysiologic measure that uses frequency-modulated stimuli to estimate audiometric thresholds across frequency regions in newborns and young children (Picton et al., 2003). Beyond threshold estimation, subcortical responses can assess the fidelity with which the auditory system encodes the temporal and spectrotemporal structure of a stimulus. The envelope following response (EFR), a class of steady-state response, tracks the temporal envelope of an amplitude-modulated stimulus and provides an objective measure of temporal coding. Dimitrijevic et al. (2016) reported that in NH listeners EFR detection thresholds corresponded to behavioral amplitude modulation detection thresholds and that EFR strength declined with aging, paralleling established age-related temporal processing deficits. The speech-evoked ABR extends this approach by using a brief synthetic syllable such as /da/, yielding both the classic neural waveforms of ABR and frequency-following components that reflect encoding of the syllable’s spectrotemporal features. Background noise can be added to the stimulus of speech-evoked ABR, implementing a complication similar to that of speech-in-noise testing. Parbery-Clark et al. (2009) found that NH musicians, who outperformed NH non-musicians on the Hearing in Noise Test, also showed more robust stimulus-to-response correlations for speech-evoked ABRs in noise [*t*(29) = 2.836, *p* = 0.008]. In a hearing aid context, Kim et al. (2025) found that in HA users digital noise reduction improved the fidelity of subcortical speech encoding, with response-to-response correlations higher in the noise-reduction condition than in the unprocessed noise condition [*t*(25) = −3.93, *p* < 0.001].

At the cortical level, the cortical auditory evoked potential (CAEP), characterized by the P1-N1-P2 complex, reflects sound processing in the auditory cortex and has been used to evaluate CI effectiveness and track cortical maturation following implantation (Sharma et al., 2002, 2015). In adults, the N1-P2 has also been related directly to speech recognition. In a large cohort of cochlear implant users, Berger et al. (2023) found that the amplitude of the N1-P2 complex in response to a target word predicted single-word recognition (*r* = 0.33, *p* < 0.001), an association that persisted after accounting for age and residual low-frequency hearing. The mismatch negativity (MMN) is elicited by embedding a deviant stimulus within a sequence of standards. As a pre-attentive response, it is generated automatically regardless of attention and is therefore measurable in infants and others who cannot follow task instructions. Rahne et al. (2010) utilized MMN as a neurophysiologic correlate to the behavioral outcomes of their logatome test. The P300, by contrast, is a cognitive event-related potential requiring active attention and stimulus categorization. A related cortical measure, the auditory change complex (ACC), is elicited by an acoustic change within an ongoing stimulus and can be evoked by transitions between spectrotemporally modulated and unmodulated sounds. This property has been used to construct an electrophysiologic counterpart to a behavioral language-agnostic test. The electrophysiologic extension of the ACT introduced earlier relies on the ACC. Simonsen et al. (2025) used the ACC to construct an electrophysiologic version of the ACT, the “E-ACT,” by alternating modulated targets with unmodulated references at the same modulation rates as the behavioral test so that the target’s appearance evokes a cortical response. They established the stimulus configuration that most reliably elicits the ACC. Because the ACC requires no behavioral response, the approach is intended to extend contrast-detection assessment to infants, young children, those with intellectual differences, and other patients who cannot perform the button-press task. In a subsequent clinical evaluation, Simonsen et al. (2026) reported moderate test-retest reliability (*I*^2^ = 0.62, *p* < 0.001) and significant but modest associations with both the behavioral ACT (*R*^2^ = 0.18, *p* < 0.001) and aided speech-in-noise reception (*R*^2^ = 0.09, *p* = 0.04). The measure remains at an early, proof-of-concept stage. Thus far, it has been studied only in adults, its reliability and predictive power lag the behavioral ACT, and it has not entered clinical use.

These measures are most useful in a few contexts: newborn hearing screening, populations for whom behavioral testing is not feasible (e.g., neonates, prelingual children, and individuals with severe cognitive or motor impairments), and for investigating neurophysiological correlates to behavioral outcomes (as seen in Rahne et al., 2010). Beyond this, however, they are limited in their clinical utility. As clinically deployed, most electrophysiologic tests measure neural integrity rather than perceptual resolution, confirming that a signal is conducted rather than quantifying how precisely it is resolved, and their interpretation is correspondingly coarse, operating closer to a present/absent determination than along the stratified performance distributions that characterize behavioral LAHTs. This coarseness can be refined, however, such as in the E-ACT which estimates a continuous contrast-detection threshold rather than a categorical response or by comparing neural responses within the same subject (Kim et al., 2025; Parbery-Clark et al., 2009; Simonsen et al., 2025). A further constraint is testing time. Electroencephalogram measures require many stimulus repetitions for signal averaging, and because the modulated and speech stimuli used in EFR, speech-evoked ABR, and ACC paradigms are considerably longer than the brief clicks used in conventional ABR, a single recording can take substantially longer than a behavioral speech recognition test (e.g., 45 min in Kim et al., 2025).

For these reasons, electrophysiologic measures occupy a distinct position within the LAHT taxonomy. They remain the most viable means of evaluating auditory system integrity and tracking the trajectory of rehabilitation when behavioral assessment falls short or is not possible.

## 5 Conclusion

**FIGURE 7:**
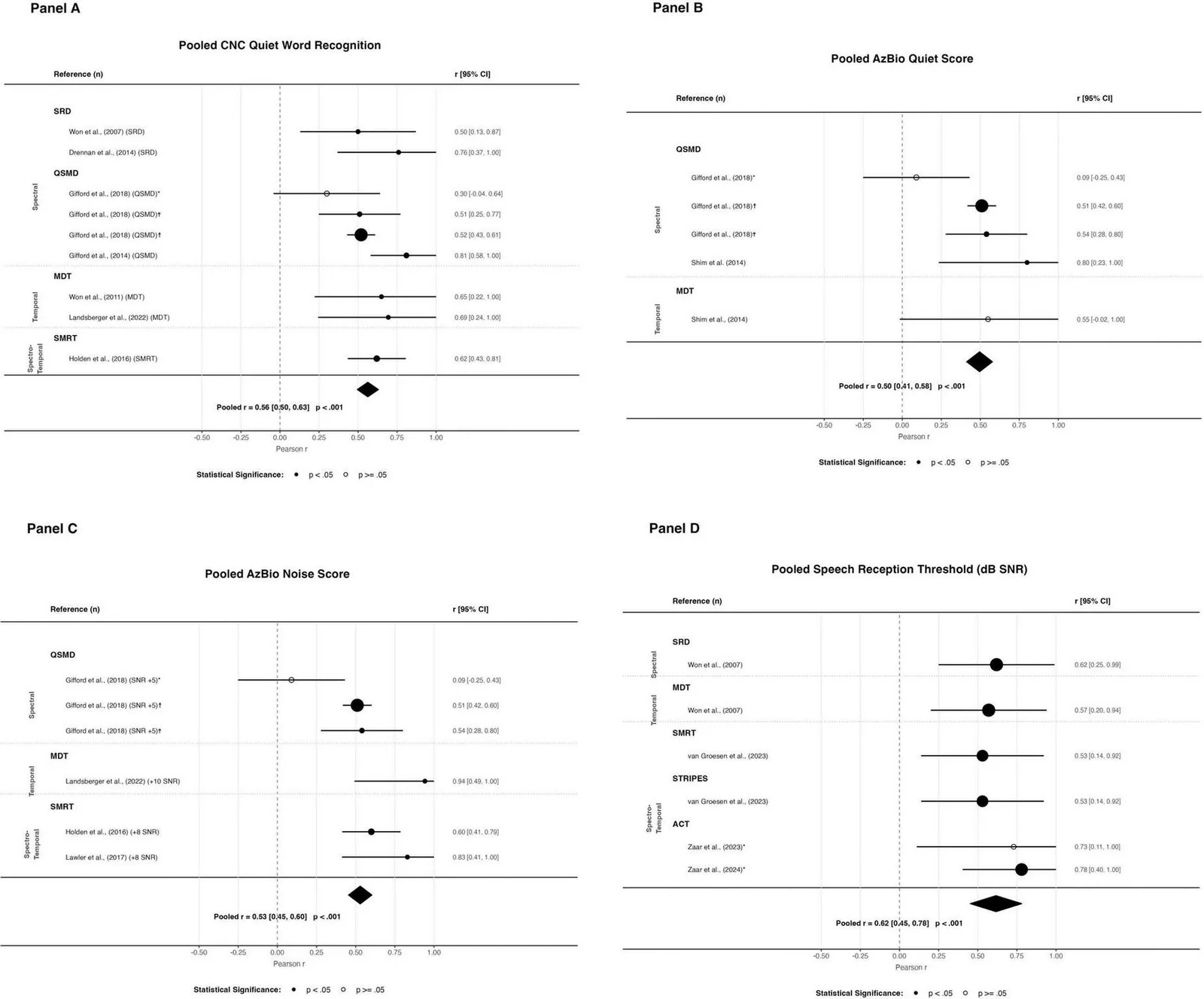
Forest PLOT. Weighted correlations between psychoacoustic resolution measures and speech recognition outcomes across CI users, HI listeners, and NH listeners using 4 outcomes: CNC Quiet word recognition **(A)**, AzBio sentence recognition in quiet **(B)**, AzBio sentence recognition in Noise **(C)**, and speech reception threshold (SRT) in noise **(D)**. Correlations for SRT and temporal modulation detection measures are presented as absolute values to facilitate comparison across outcome measures. Point size reflects sample size weighting. Open circles indicate non-significant correlations (*p* ≥ 0.05). Dashed vertical line denotes *r* = 0. Dotted horizontal lines demarcate subdomain boundaries. Gifford et al. (2018) QSMD data in **(A–C)** are stratified by implantation group: prelingually deafened children (*), prelingually deafened adults (‡), and postlingually deafened adults (†).

Taken together, the literature reviewed here supports that language agnostic hearing tests can measure dimensions of auditory function that speech-based assessment is unable to access. Spectral and temporal resolution measures isolate the integrity of peripheral encoding with a mechanistic specificity that word and sentence scores cannot. Figure 7 illustrates the correlational analyses reported across psychoacoustic resolution studies reviewed here.

Naturalistic tests offer unique measures of CI outcomes beyond language intelligibility by exploring the discrete acoustic units that underlie it, environmental sound recognition, ecological engagement, and quality of life. None of these tools displace speech testing, nor should they, but together they begin to address dimensions of auditory function that speech testing alone cannot capture. Table 3 summarizes correlational findings for the studies reviewed here. The field now has a sufficiently developed set of complementary measures to start integrating them into both research design and clinical practice—-a step that the ACT has already demonstrated as feasible.

**TABLE 3:** Additional summary of language agnostic hearing tests and correlations with speech measures.

Domain	Sub-domain	Test	Publication	Speech in quiet (Pearson *r*, *p*-value)	Speech in noise (Pearson r)
Psychoacoustic	Spectral	SRD	Anderson et al. (2011)	IEEE: (0.69‡, 0.005)^4^	N/A
			Shim et al. (2014)	Unaided W-22: (0.74, < 0.001)	N/A
			Dai et al. (2018)	Mandarin Word: (0.84, < 0.001†) Mandarin Sentence: (0.537, 0.003)^1^	N/A
	Temporal	MDT/Easy MDT	Landsberger and Stupak (2022)	Consonant Identification: (−0.821, 0.007†)	N/A
			Shim et al. (2014)	Unaided W-22: (−0.52, < 0.05)	N/A
	Spectrotemporal	SMRT	Jeddi et al. (2019)	Sentence Score——: (0.556, < 0.001) WRS——: (0.573, < 0.001)	N/A
			Holden et al. (2016)	N/A	HINT: (−0.48, < .005)
		STRIPES	Goehring et al. (2019)	N/A	BKB: (−0.59,.016)
		STM Detection	Choi et al. (2016)	CVC: (−0.821, < 0.001†) K-CID: (−0.762, < 0.001†)	N/A
		ACT	Zaar et al. (2023)	N/A	SSN: (0.59,.018†)
Naturalistic	Phoneme/Logatome	Logatome	Rahne et al. (2010)	N/A	
		LIT-AD	Ching et al. (2021)	N/A	BKB-Like: (−0.73, < .001)
	Environmental Sounds	FEST-I	Harris et al. (2017)	IEEE: (0.752, < 0.001) PRESTO: (0.795, < 0.001) CID-W22: (0.762, < 0.001)	N/A
	Music	CAMP/Music	N/A	N/A	N/A

‡Values are Pearson’s r converted from reported R^2^. The sign of r was assigned based on the reported direction of the association. §MDT and EasyMDT measure the minimum change in modulation frequency; a lower score indicates higher temporal resolution. || Jeddi et al. (2019) used two lists of 25 Persian-language phonetically balanced monosyllabic words and eight list pairs containing everyday Persian-language sentences presented orally. ACT, Audible Contrast Threshold; CAMP, Clinical Appreciation of Music Perception; FEST-I, Familiar Environmental Sound Test–Identification; IEEE, Institute of Electrical and Electronics Engineers; LIT-AD, Language-Independent Test of Auditory Discrimination; MDT/EasyMDT: Modulation Detection Threshold/Easy Modulation Detection Threshold; QSMD/EasyQSMD, Quick Spectral Modulation Detection/Easy Quick Spectral Modulation Detection; SMRT, Spectral-Temporally Modulated Ripple Test; SRD, Spectral Ripple Discrimination; STM, Spectrotemporal Modulation; STRIPES, Spectrotemporal Ripple for Investigating Processor EffectivenesS.

The central theme of this review is one of cautious optimism: that LAHTs are positioned to be productive complements to standard tests, not replacements. LAHTs measure dimensions of auditory function that speech-based assessment cannot, yet the evidence does not support their use as a substitute for speech testing.

### 5.1 “A correlate does not a surrogate make”

The correlations between language agnostic measures and speech recognition outcomes are robust and consistent across the literature, but they do not establish these tests as surrogates for speech assessment (Fleming and DeMets, 1996). Drennan et al. (2014) cautioned explicitly against that inference for spectral ripple discrimination, and the caution applies broadly: a measure that predicts speech scores is not the same as a measure that replaces them. This is particularly salient when we cannot consistently characterize the relationship between any CI hearing test and patient-reported quality of life measures. That gap between CI performance and lived benefit is among the most important unresolved questions the field faces.

The aliasing findings complicate the picture further. If spectral resolution carries meaningful clinical weight only up to 2 or 3 RPO, and marginal gains above that threshold do not yield returns for real world performance, then a dimension that has received sustained research attention may not be the most productive one to optimize. What cue, or combination of cues, represents the most promising target for improving CI outcomes remains an open question—and an important one.

The pediatric literature raises a related uncertainty. Children with cochlear implants underperform adults on spectral and spectrotemporal threshold measures despite achieving comparable or superior speech recognition scores. Superior temporal sensitivity may be the explanation, however neither spectral nor temporal resolution has yet demonstrated a significant correlation with speech outcomes in this population. What these findings do establish, and this is itself a contribution of LAHTs, is that prelingually deafened CI users appear to organize auditory perception around cues that current measures have not yet fully characterized.

Logatome and phoneme discrimination tasks represent perhaps the most underdeveloped category of LAHTs reviewed here. Rahne et al. (2010) demonstrated that a logatome discrimination paradigm afforded substantially greater resolution across the CI performance range than a standard sentence test, and Ching et al. (2021) showed that their phoneme-based LIT-AD correlated significantly with sentence perception in both HA and CI users. However, both studies relied on small samples, were conducted in a single language, and have not been replicated or validated in clinical settings. This category of testing, occupying a theoretically important middle ground between purely acoustic measures and conventional speech testing, warrants further attention. Cross-linguistic phoneme selection, adaptive test implementations, and prospective pre- and post-implant validation studies can help establish these LAHTs as clinically informative tools. Finally, the evidence base across all domains of LAHTs is constrained by small sample sizes—-the median sample size in this review was 40 listeners—-and limited test standardization.

### 5.2 What the future holds

LAHTs offer three promising possibilities to the field of hearing assessment.

The first is as a clinical tool. Several tests reviewed here have demonstrated predictive value for CI candidacy through a range of approaches. Unaided spectral and spectrotemporal resolution measures have shown strong alignment with the aided sentence criterion independent of language proficiency (Shim et al., 2014; Choi et al., 2016), and phoneme discrimination measures have been applied to estimate the probability that a HA user would achieve better speech perception with a CI (Ching et al., 2021). The shared appeal of these approaches is not greater accuracy than the aided sentence test, but the removal of its logistical and linguistic costs. Psychoacoustic and phoneme discrimination tasks are administered unaided, are language-independent, and show minimal learning effects across repeated administration, making them promising screening tools particularly for linguistically diverse patients. Beyond candidacy, the ACT is already in clinical use to guide hearing aid fitting configuration (Zaar et al., 2024b), and spectral modulation detection measures can be applied to inform bimodal fitting decisions (Kessler et al., 2020). More broadly, psychoacoustic measures correlate with speech recognition outcomes across the spectral, temporal, and spectrotemporal domains, offering a framework for characterizing the trajectory of CI outcomes over time. Phoneme testing occupies a distinct position within this landscape: by using the building blocks of language, these tests retain sensitivity to the phonemic cues underlying speech perception while stripping away the lexical and syntactic context that confounds language-based performance. That said, the clinical translation of these measures remains incomplete. The candidacy evidence to date rests on small, predominantly single-language and single-site samples, and prospective validation has yet to be conducted. Should such validation take place, these measures could meaningfully expand the clinical toolkit for CI assessment, particularly for linguistically diverse populations currently underserved by speech-based testing.

LAHTs also offer a mechanistic precision befitting a research tool. Because LAHTs isolate specific acoustic cues, they can be used to investigate the factors underlying variability in HA and CI outcomes in ways that speech scores cannot, such as isolating working memory from auditory function or disentangling audibility from distortion (Bernstein et al., 2013; O’Neill et al., 2019). DeFreese et al. (2024) used spectrotemporal measures to evaluate the relationship between modiolar distance and auditory resolution, and the dissociation between MDT and SMRT performance reported by Landsberger and Stupak (2022) provided evidence that temporal and spectral processing reflect distinct auditory mechanisms. LAHTs have also served as outcome measures in CI engineering research, enabling systematic evaluation of how device parameters (e.g., electrode configuration, processing strategy, channel number) translate into perceptual gains (Jones et al., 2013; Park et al., 2015; Won et al., 2007, 2011). These measures allow us to understand why implants work differently across patients, and what can be done about it.

The third possibility is creating a more equitable landscape for hearing health in the CI population as well as the hearing impaired population more broadly. For patients with LEP, LAHTs remove the linguistic barrier embedded in clinical practice. For populations whose access to conventional speech batteries is limited, whether by clinical resources, geography, or patient characteristics, LAHTs offer a practical alternative. Psychoacoustic tests have been validated and are administrable in under 10 min, require minimal to no additional equipment, and can integrate into existing clinical workflows. STRIPES has even demonstrated feasibility over telehealth visits (Archer-Boyd et al., 2023). These tests are valid across language groups, such as Farsi- and Dutch-speaking CI populations (Jeddi et al., 2019; van Groesen et al., 2023). The equity argument also extends beyond linguistic diversity to populations for whom verbal response formats are inaccessible: patients with motor speech disorders, tracheostomy dependence, non-speaking autism spectrum disorder, cognitive impairment, and prelingual deafness all face assessment barriers that LAHTs are positioned to address.

No matter how promising a test may appear, it raises the question that defines both the value and the limits of these measures: how fully can auditory function be disentangled from the linguistic and cognitive systems it serves? Speech perception is not a modular output of the cochlea; it is the emergent property of a system in which peripheral encoding, phonological processing, working memory, and linguistic inference interact in ways that transcend any single score. Every behavioral measure reviewed here retains some degree of this interdependence: psychoacoustic tasks still require attention, working memory, and procedural learning, and logatome- and phoneme-based tests remain tied to the phonotactic structure of a particular language. Although LAHTs cannot fully disentangle these systems, they can accomplish a narrower but meaningful objective. By removing the lexical, syntactic, and semantic demands that speech materials impose, they shift the locus of measurement toward auditory encoding and away from higher-order language processing. The dissociation reported by O’Neill et al. (2019), in which working memory correlated with speech recognition but not with spectral resolution, illustrates this. A psychoacoustic measure can target auditory function with a degree of independence from cognitive load that speech scores cannot.

The contribution of LAHTs is therefore one of degrees. Rather than resolving the auditory, linguistic, and cognitive components of speech perception into fully separable parts, LAHTs can be positioned along a continuum from the most language-laden speech materials to the most acoustically constrained electrophysiologic measures. The mechanistic questions that most matter for CI outcomes, such as why two patients with similar audiometric profiles achieve divergent results, what cues they rely on, and which components of their auditory system are most amenable to intervention, are intrinsically multidimensional. LAHTs offer a unique perspective on a complex issue by distinguishing more cleanly than speech testing what a patient can hear from what they can understand. Full realization of the utility of these tests will require multidisciplinary, multidimensional assessment that integrates standard testing with non-linguistic measures, cognitive evaluation, and patient-reported outcomes.

The question of how we measure success remains incompletely answered. While the universal goal posts continue to be speech recognition scores, we must also concede that these are not appropriate surrogates for quality of life, and the relationship between non-linguistic hearing measures and patient-reported outcomes has not yet been systematically investigated (McRackan et al., 2018). That gap is among the most consequential open questions the field faces: if non-linguistic measures predict speech scores, and speech scores do not predict quality of life, then the chain connecting what we measure in the clinic to what patients experience remains broken at its most important link. The work reviewed here suggests that LAHTs are valuable research tools and ready to be considered seriously as complements to standard clinical batteries. Whether they can help close that gap between measurable performance and lived benefit will depend on the continued efforts of those forging the field.

## References

[B1] Anderson E. S. Nelson D. A. Kreft H. Nelson P. B. Oxenham A. J. (2011). Comparing spatial tuning curves, spectral ripple resolution, and speech perception in cochlear implant users. *J. Acoust. Soc. Am.* 130 364–375. 10.1121/1.3589255 21786905 PMC3155592

[B2] Anderson E. S. Oxenham A. J. Nelson P. B. Nelson D. A. (2012). Assessing the role of spectral and intensity cues in spectral ripple detection and discrimination in cochlear-implant users. *J. Acoust. Soc. Am.* 132 3925–3934. 10.1121/1.4763999 23231122 PMC3529540

[B3] Archer-Boyd A. W. Goehring T. Carlyon R. P. (2020). The effect of free-field presentation and processing strategy on a measure of spectro-temporal processing by cochlear-implant listeners. *Trends Hear.* 24:2331216520964281. 10.1177/2331216520964281 33305696 PMC7734493

[B4] Archer-Boyd A. W. Harland A. Goehring T. Carlyon R. P. (2023). An online implementation of a measure of spectro-temporal processing by cochlear-implant listeners. *JASA Exp. Lett.* 3:014402. 10.1121/10.0016838 36725534

[B5] Archer-Boyd A. W. Southwell R. V. Deeks J. M. Turner R. E. Carlyon R. P. (2018). Development and validation of a spectro-temporal processing test for cochlear-implant listeners. *J. Acoust. Soc. Am.* 144 2983–2997. 10.1121/1.5079636 30522311 PMC6805218

[B6] Aronoff J. M. Landsberger D. M. (2013). The development of a modified spectral ripple test. *J. Acoust. Soc. Am.* 134 EL217–EL222. 10.1121/1.4813802 23927228 PMC3732300

[B7] Bacon S. P. Viemeister N. F. (1985). Temporal modulation transfer functions in normal-hearing and hearing-impaired listeners. *Audiology* 24 117–134. 10.3109/00206098509081545 3994589

[B8] Batalova J. Zong J. (2016). *Language Diversity and English Proficiency in the United States.* Washington, D.C: Migrationpolicy.Org.

[B9] Berg K. A. Noble J. H. Dawant B. M. Dwyer R. T. Labadie R. F. Gifford R. H. (2019). Speech recognition as a function of the number of channels in perimodiolar electrode recipients. *J. Acoust. Soc. Am.* 145 1556–1564. 10.1121/1.5092350 31067952 PMC6435372

[B10] Berg K. A. Noble J. H. Dawant B. M. Dwyer R. T. Labadie R. F. Gifford R. H. (2020). Speech recognition with cochlear implants as a function of the number of channels: Effects of electrode placement. *J. Acoust. Soc. Am.* 147:3646. 10.1121/10.0001316 32486813 PMC7255811

[B11] Bergeron F. Berland A. Fitzpatrick E. M. Vincent C. Giasson A. Leung Kam K. et al. (2019). Development and validation of the FrBio, an international French adaptation of the AzBio sentence lists. *Intern. J. Audiol.* 58 510–515. 10.1080/14992027.2019.1581950 31074295

[B12] Berger J. I. Gander P. E. Kim S. Schwalje A. T. Woo J. Na Y. et al. (2023). Neural correlates of individual differences in speech-in-noise performance in a large cohort of cochlear implant users. *Ear and Hearing* 44, 1107–1120. 10.1097/AUD.0000000000001357 37144890 PMC10426791

[B13] Bernstein J. G. W. Danielsson H. Hällgren M. Stenfelt S. Rönnberg J. Lunner T. (2016). Spectrotemporal modulation sensitivity as a predictor of speech-reception performance in noise with hearing aids. *Trends Hear.* 20:2331216516670387. 10.1177/2331216516670387 27815546 PMC5098798

[B14] Bernstein J. G. W. Mehraei G. Shamma S. Gallun F. J. Theodoroff S. M. Leek M. R. (2013). Spectrotemporal modulation sensitivity as a predictor of speech intelligibility for hearing-impaired listeners. *J. Am. Acad. Audiol.* 24 293–306. 10.3766/jaaa.24.4.5 23636210 PMC3973426

[B15] Brochier T. McKay C. McDermott H. (2018). Rate modulation detection thresholds for cochlear implant users. *J. Acoust. Soc. Am.* 143:1214. 10.1121/1.5025048 29495682

[B16] Brown C. J. Abbas P. J. Fryauf-Bertschy H. Kelsay D. Gantz B. J. (1994). Intraoperative and postoperative electrically evoked auditory brain stem responses in nucleus cochlear implant users: Implications for the fitting process. *Ear Hear.* 15 168–176. 10.1097/00003446-199404000-00006 8020649

[B17] Bysouth-Young D. Guérit F. Shahidi L. Carlyon R. P. (2025). Measurement of spectro-temporal processing by cochlear implant users: Effects of stimulus level and validation of an online implementation. *Ear Hear.* 46:1417. 10.1097/AUD.0000000000001676 40474344 PMC12533785

[B18] Ching T. Y. C. Dillon H. Hou S. Seeto M. Sodan A. Chong-White N. (2021). Development and evaluation of a language-independent test of auditory discrimination for referrals for cochlear implant candidacy assessment. *Ear Hear.* 43 1151–1163. 10.1097/AUD.0000000000001166 34812793 PMC9197147

[B19] Choi J. E. Hong S. H. Won J. H. Park H.-S. Cho Y. S. Chung W.-H. et al. (2016). Evaluation of cochlear implant candidates using a non-linguistic spectrotemporal modulation detection test. *Sci. Rep.* 6:35235. 10.1038/srep35235 27731425 PMC5059668

[B20] Choi J. S. Adams M. E. Crimmins E. M. Lin F. R. Ailshire J. A. (2024). Association between hearing aid use and mortality in adults with hearing loss in the USA: A mortality follow-up study of a cross-sectional cohort. *Lancet Healthy Long.* 5 e66–e75. 10.1016/S2666-7568(23)00232-5 38183998 PMC11501007

[B21] Commitee on Meaningful Outcome Measures in Adult Hearing Health Care, Board on Health Care Services, Health and Medicine Division, and National Academies of Sciences, Engineering, and Medicine (2025). Measuring Meaningful Outcomes for Adult Hearing Health Interventions. (Washington, D.C: National Academies Press). 40354504

[B22] Dai C. Zhao Z. Shen W. Zhang D. Lei G. Qiao Y. et al. (2018). Evaluation of Mandarin Chinese speech recognition in adults with cochlear implants using the spectral ripple discrimination test. *Med. Sci. Monit.* 24 3557–3563. 10.12659/MSM.907491 29806954 PMC6001366

[B23] Davidson L. S. Geers A. E. Uchanski R. M. (2021). Spectral modulation detection performance and speech perception in pediatric cochlear implant recipients. *Am. J. Audiol.* 30 1076–1087. 10.1044/2021_AJA-21-00076 34670098 PMC9126113

[B24] Davies-Venn E. Nelson P. Souza P. (2015). Comparing auditory filter bandwidths, spectral ripple modulation detection, spectral ripple discrimination, and speech recognition: Normal and impaired hearing. *J. Acoust. Soc. Am.* 138 492–503. 10.1121/1.4922700 26233047 PMC4514721

[B25] DeFreese A. Camarata S. Sunderhaus L. Holder J. Berg K. Lighterink M. et al. (2024). The impact of spectral and temporal processing on speech recognition in children with cochlear implants. *Sci. Rep.* 14:14094. 10.1038/s41598-024-63932-w 38890428 PMC11189542

[B26] Dimitrijevic A. Alsamri J. John M. S. Purcell D. George S. Zeng F.-G. (2016). Human envelope following responses to amplitude modulation: Effects of aging and modulation depth. *Ear Hear.* 37 e322–e335. 10.1097/AUD.0000000000000324 27556365 PMC5031488

[B27] Dornhoffer J. R. Reddy P. Meyer T. A. Schvartz-Leyzac K. C. Dubno J. R. McRackan T. R. (2021). Individual differences in speech recognition changes after cochlear implantation. *JAMA Otolaryngology–Head Neck Surg.* 147 280–286. 10.1001/jamaoto.2020.5094 33410869 PMC7791403

[B28] Drennan W. R. Anderson E. S. Won J. H. Rubinstein J. T. (2014). Validation of a clinical assessment of spectral-ripple resolution for cochlear implant users. *Ear Hear.* 35:e92. 10.1097/AUD.0000000000000009 24552679 PMC3999174

[B29] Duquesnoy A. J. (1983). The intelligibility of sentences in quiet and in noise in aged listeners. *J. Acoust. Soc. Am.* 74 1136–1144. 10.1121/1.390037 6643835

[B30] Elgaddal N. Weeks J. D. (2023). Percentage of adults Aged =45 Years who use a hearing Aid,† by sex and age group—national health interview survey, United States, 2021§. *Morbidity Mortal. Weekly Rep. (MMWR)* 72:163. 10.15585/mmwr.mm7206a5 36757858 PMC9925135

[B31] Erb J. Ludwig A. A. Kunke D. Fuchs M. Obleser J. (2019). Temporal sensitivity measured shortly after cochlear implantation predicts 6-month speech recognition outcome. *Ear Hear.* 40:27. 10.1097/AUD.0000000000000588 29697465

[B32] Fleming T. R. DeMets D. L. (1996). Surrogate end points in clinical trials: Are we being misled? *Ann. Intern. Med.* 125, 605–613. 10.7326/0003-4819-125-7-199610010-00011 8815760

[B33] Friesen L. M. Shannon R. V. Baskent D. Wang X. (2001). Speech recognition in noise as a function of the number of spectral channels: Comparison of acoustic hearing and cochlear implants. *J. Acoust. Soc. Am.* 110 1150–1163. 10.1121/1.1381538 11519582

[B34] Gifford R. H. Hedley-Williams A. Spahr A. J. (2014). Clinical assessment of spectral modulation detection for adult cochlear implant recipients: A non-language based measure of performance outcomes. *Intern. J. Audiol.* 53 159–164. 10.3109/14992027.2013.851800 24456178 PMC4067975

[B35] Gifford R. H. Noble J. H. Camarata S. M. Sunderhaus L. W. Dwyer R. T. Dawant B. M. et al. (2018). The relationship between spectral modulation detection and speech recognition: Adult versus pediatric cochlear implant recipients. *Trends Hear.* 22:2331216518771176. 10.1177/2331216518771176 29716437 PMC5949922

[B36] Goehring T. Archer-Boyd A. Deeks J. M. Arenberg J. G. Carlyon R. P. (2019). A site-selection strategy based on polarity sensitivity for cochlear implants: Effects on spectro-temporal resolution and speech perception. *J. Assoc. Res. Otolaryngol.* 20, 431–448. 10.1007/s10162-019-00724-4 31161338 PMC6646483

[B37] Govindan A. Singer A. Zekavat L. Jia T. Wong K. Kuang J. et al. (2025). Clinician perspectives on the management of hearing loss in patients with limited english proficiency. *Otolaryngology–Head Neck Surg.* 172 1232–1241. 10.1002/ohn.1089 39724293

[B38] Grant K. W. Walden T. C. (2013). Understanding excessive SNR loss in hearing-impaired listeners. *J. Am. Acad. Audiol.* 24 258–273. 10.3766/jaaa.24.4.3 23636208

[B39] Greiner R. C. Rubinstein J. T. Kohlberg G. D. (2023). Investigating socioeconomic barriers to cochlear implantation. *Otol. Neurotol.* 44:e660. 10.1097/MAO.0000000000003985 37604510

[B40] Hamel B. L. Vasil K. Shafiro V. Moberly A. C. Harris M. S. (2020). Safety-relevant environmental sound identification in cochlear implant candidates and users. *Laryngoscope* 130 1547–1551. 10.1002/lary.28285 31498464

[B41] Harris M. S. Boyce L. Pisoni D. B. Shafiro V. Moberly A. C. (2017). The relationship between environmental sound awareness and speech recognition skills in experienced cochlear implant users. *Otol. Neurotol.* 38:e308. 10.1097/MAO.0000000000001514 28731964 PMC6205294

[B42] Henry B. A. Turner C. W. (2003). The resolution of complex spectral patterns by cochlear implant and normal-hearing listeners. *J. Acoust. Soc. Am.* 113 2861–2873. 10.1121/1.1561900 12765402

[B43] Henry B. A. Turner C. W. Behrens A. (2005). Spectral peak resolution and speech recognition in quiet: Normal hearing, hearing impaired, and cochlear implant listeners. *J. Acoust. Soc. Am.* 118 1111–1121. 10.1121/1.1944567 16158665

[B44] Holden L. K. Firszt J. B. Reeder R. M. Uchanski R. M. Dwyer N. Y. Holden T. A. (2016). Factors affecting outcomes in cochlear implant recipients implanted with a perimodiolar electrode array located in scala tympani. *Otol. Neurotol.* 37:1662. 10.1097/MAO.0000000000001241 27755365 PMC5113723

[B45] Hwa T. P. Wen C. Z. Ruckenstein M. J. (2021). Assessment of music experience after cochlear implantation: A review of current tools and their utilization. *World J. Otorhinolaryngol. Head Neck Surg.* 7 116–125. 10.1016/j.wjorl.2021.02.003 33997721 PMC8103528

[B46] Jeddi Z. Lotfi Y. Moossavi A. Bakhshi E. Hashemi S. B. (2019). Correlation between auditory spectral resolution and speech perception in children with cochlear implants. *Iran. J. Med. Sci.* 44 382–389. 10.30476/IJMS.2019.44967 31582862 PMC6754529

[B47] Joint Committee on Infant Hearing (2019). Year 2019 position statement: Principles and guidelines for early hearing detection and intervention programs. *J. Early Hear. Detect. Intervent.* 4 1–44. 10.15142/FPTK-B748

[B48] Jones G. L. Ho Won J. Drennan W. R. Rubinstein J. T. (2013). Relationship between channel interaction and spectral-ripple discrimination in cochlear implant users. *J. Acoust. Soc. Am.* 133 425–433. 10.1121/1.4768881 23297914 PMC3548834

[B49] Jung C. Shah K. V. Ferraro T. Bigelow D. C. Ruckenstein M. J. Peng-Hwa T. (2026). Non-English validation of the AzBio sentence test: A systematic review of current applications and early adoption patterns. *Otol. Neurotol.* 47:e83. 10.1097/MAO.0000000000004663 41145396

[B50] Kessler D. M. Wolfe J. Blanchard M. Gifford R. H. (2020). Clinical application of spectral modulation detection: Speech recognition benefit for combining a cochlear implant and contralateral hearing aid. *J. Speech Lang. Hear. Res. JSLHR* 63 1561–1571. 10.1044/2020_JSLHR-19-00304 32379527 PMC7842114

[B51] Killion M. C. Niquette P. A. (2000). What can the pure-tone audiogram tell us about a patient’s SNR loss? *Hear. J.* 53 52–53. 10.1097/00025572-200003000-00006

[B52] Killion M. C. Niquette P. A. Gudmundsen G. I. Revit L. J. Banerjee S. (2004). Development of a quick speech-in-noise test for measuring signal-to-noise ratio loss in normal-hearing and hearing-impaired listeners. *J. Acoust. Soc. Am.* 116 2395–2405. 10.1121/1.1784440 15532670

[B53] Kim S. Y. Min C. Kim H.-J. Lee C. H. Sim S. Park B. et al. (2020). Mortality and cause of death in hearing loss participants: A longitudinal follow-up study using a national sample cohort. [Miscellaneous Article]. *Otol. Neurotol.* 41 25–32. 10.1097/MAO.0000000000002429 31634278

[B54] Kim S. Schroeder M. Bharadwaj H. M. (2025). Effect of digital noise reduction processing on subcortical speech encoding and relationship to behavioral outcomes. *Sci. Rep.* 15:22198. 10.1038/s41598-025-07652-9 40594752 PMC12219303

[B55] Koch D. B. Downing M. Osberger M. J. Litvak L. (2007). Using current steering to increase spectral resolution in cii and hires 90K users. *Ear Hear.* 28:38S. 10.1097/AUD.0b013e31803150de 17496643

[B56] Kwak M. Y. Kang Y. K. Kim D. H. An Y.-H. Shim H. J. (2018). Further beneficial effect of hearing aids on speech recognition performance besides amplification: Importance of the restoration of symmetric hearing. *Otol. Neurotol.* 39:e618. 10.1097/MAO.0000000000001928 30113555

[B57] Landsberger D. M. Stupak N. (2022). Evaluation of a tool for measuring temporal modulation detection. *Ear Hear.* 43:448. 10.1097/AUD.0000000000001107 34380982

[B58] Landsberger D. M. Dwyer R. T. Stupak N. Gifford R. H. (2019a). Validating a quick spectral modulation detection task. *Ear Hear.* 40 1478–1480. 10.1097/AUD.0000000000000713 31033635 PMC6776705

[B59] Landsberger D. M. Padilla M. Martinez A. S. Eisenberg L. S. (2018). Spectral-Temporal modulated ripple discrimination by children with cochlear implants. *Ear Hear.* 39 60–68. 10.1097/AUD.0000000000000463 28682810 PMC8905526

[B60] Landsberger D. M. Stupak N. Green J. Tona K. Padilla M. Martinez A. S. et al. (2019b). Temporal modulation detection in children and adults with cochlear implants: Initial results. *Otol. Neurotol.* 40:e311. 10.1097/MAO.0000000000002122 30741912

[B61] Lawler M. Yu J. Aronoff J. M. (2017). Comparison of the spectral-temporally modulated ripple test with the arizona biomedical institute sentence test in cochlear implant users. *Ear Hear.* 38 760–766. 10.1097/AUD.0000000000000496 28957975 PMC5741801

[B62] Looi V. Gfeller K. Driscoll V. (2012). Music appreciation and training for cochlear implant recipients: A review. *Sem. Hear.* 33 307–334. 10.1055/s-0032-1329222 23459244 PMC3583543

[B63] Luzum N. R. Hamel B. L. Shafiro V. Harris M. S. (2023). Identification accuracy of safety-relevant environmental sounds in adult cochlear implant users. *Laryngoscope* 133 2388–2393. 10.1002/lary.30475 36317721 PMC10149563

[B64] McMahon K. R. Moberly A. C. Shafiro V. Harris M. S. (2018). Environmental sound awareness in experienced cochlear implant users and cochlear implant candidates. *Otol. Neurotol.* 39 e964–e971. 10.1097/MAO.0000000000002006 30252797 PMC6242748

[B65] McRackan T. R. Bauschard M. Hatch J. L. Franko-Tobin E. Droghini H. R. Nguyen S. A. et al. (2018). Meta-analysis of quality of life improvement after cochlear implantation and associations with speech recognition abilities. *Laryngoscope* 128 982–990. 10.1002/lary.26738 28731538 PMC5776066

[B66] Messersmith J. J. Entwisle L. Warren S. Scott M. (2019). Clinical practice guidelines: Cochlear implants. *J. Am. Acad. Audiol.* 30 827–844. 10.3766/jaaa.19088 31823835

[B67] Mirza S. Douglas S. A. Lindsey P. Hildreth T. Hawthorne M. (2003). Appreciation of music in adult patients with cochlear implants: A patient questionnaire. *Cochlear Implants Intern.* 4 85–95. 10.1179/cim.2003.4.2.85 18792140

[B68] Moberly A. C. Castellanos I. Mattingly J. K. (2018a). Neurocognitive factors contributing to cochlear implant candidacy. *Otol. Neurotol.* 39:e1010. 10.1097/MAO.0000000000002052 30444846 PMC7037805

[B69] Moberly A. C. Vasil K. J. Wucinich T. L. Safdar N. Boyce L. Roup C. et al. (2018b). How does aging affect recognition of spectrally degraded speech? *Laryngoscope* 128:10.1002/lary.27457. 10.1002/lary.27457 30325518 PMC6572764

[B70] Nilsson M. Soli S. D. Sullivan J. A. (1994). Development of the Hearing In Noise Test for the measurement of speech reception thresholds in quiet and in noise. *J. Acoust. Soc. Am.* 95 1085–1099. 10.1121/1.408469 8132902

[B71] Nimmons G. L. Kang R. S. Drennan W. R. Longnion J. Ruffin C. Worman T. et al. (2008). Clinical assessment of music perception in cochlear implant listeners. *Otol. Neurotol.* 29:149. 10.1097/mao.0b013e31812f7244 18309572 PMC2669784

[B72] O’Neill E. R. Kreft H. A. Oxenham A. J. (2019). Cognitive factors contribute to speech perception in cochlear-implant users and age-matched normal-hearing listeners under vocoded conditions. *J. Acoust. Soc. Am.* 146 195–210. 10.1121/1.5116009 31370651 PMC6637026

[B73] Outcome Measures in Adult Hearing Health Care, Board on Health Care Services, Health and Medicine Division, and National Academies of Sciences, Engineering, and Medicine (2025). *Measuring Meaningful Outcomes for Adult Hearing Health Interventions.* Washington, D.C: National Academies Press, 29104. 10.17226/29104 40354504

[B74] Parbery-Clark A. Skoe E. Kraus N. (2009). Musical experience limits the degradative effects of background noise on the neural processing of sound. *J. Neurosci.* 29 14100–14107. 10.1523/JNEUROSCI.3256-09.2009 19906958 PMC6665054

[B75] Park M.-H. Won J. H. Horn D. L. Rubinstein J. T. (2015). Acoustic temporal modulation detection in normal-hearing and cochlear implanted listeners: Effects of hearing mechanism and development. *J. Assoc. Res. Otolaryngol.* 16 389–399. 10.1007/s10162-014-0499-z 25790949 PMC4417089

[B76] Picton T. W. John M. S. Dimitrijevic A. Purcell D. (2003). Human auditory steady-state responses: Respuestas auditivas de estado estable en humanos. *Intern. J. Audiol.* 42 177–219. 10.3109/14992020309101316 12790346

[B77] Plomp R. (1986). A signal-to-noise ratio model for the speech-reception threshold of the hearing impaired. *J. Speech Lang. Hear. Res.* 29 146–154. 10.1044/jshr.2902.146 3724108

[B78] Rahne T. Ziese M. Rostalski D. Mühler R. (2010). Logatome discrimination in cochlear implant users: Subjective tests compared to the mismatch negativity. *Sci. World J.* 10 329–339. 10.1100/tsw.2010.28 20191246 PMC5763825

[B79] Rivas A. Perkins E. Rivas A. Rincon L. A. Litvak L. Spahr T. et al. (2021). Development and validation of the Spanish AzBio sentence corpus. *Otol. Neurotol.* 42:154. 10.1097/MAO.0000000000002970 33885261

[B80] Rodriguez E. R. Shader M. J. (2025). Comparing the AzBio sentence-in-noise test in english and Spanish in bilingual adults. *J. Am. Acad. Audiol.* 36 2–10. 10.3766/jaaa.230120 39933730 PMC12445270

[B81] Sanchez-Lopez R. Fereczkowski M. Neher T. Santurette S. Dau T. (2020). Robust data-driven auditory profiling towards precision audiology. *Trends Hear.* 24:2331216520973539. 10.1177/2331216520973539 33272110 PMC7720332

[B82] Sanchez-Lopez R. Zaar J. Laugesen S. (2026). Evaluating user-operated tests of audible contrast sensitivity towards efficient hearing healthcare services. *medRxiv [Preprint]* 10.1101/2025.07.28.25332303 PMC13362146 42441667

[B83] Shafiro V. (2008a). Development of a large-item environmental sound test and the effects of short-term training with spectrally-degraded stimuli. *Ear Hear.* 29 775–790. 10.1097/AUD.0b013e31817e08ea 18596641

[B84] Shafiro V. (2008b). Identification of environmental sounds with varying spectral resolution. *Ear Hear.* 29 401–420. 10.1097/AUD.0b013e31816a0cf1 18344871

[B85] Shafiro V. Gygi B. Cheng M.-Y. Vachhani J. Mulvey M. (2011). Perception of environmental sounds by experienced cochlear implant patients. *Ear Hear.* 32 511–523. 10.1097/AUD.0b013e3182064a87 21248643 PMC3115425

[B86] Shafiro V. Luzum N. Moberly A. C. Harris M. S. (2022). Perception of environmental sounds in cochlear implant users: A systematic review. *Front. Neurosci.* 15:788899. 10.3389/fnins.2021.788899 35082595 PMC8785216

[B87] Shafiro V. Moberly A. C. Pisoni D. B. Tamati T. N. (2025). Evolving perspectives on speech perception assessment in adults with cochlear implants: Are we using the right tests? *Front. Neurosci.* 19:1667467. 10.3389/fnins.2025.1667467 41035521 PMC12479439

[B88] Sharma A. Dorman M. F. Spahr A. J. (2002). Rapid development of cortical auditory evoked potentials after early cochlear implantation. *NeuroReport* 13 1365–1368. 10.1097/00001756-200207190-00030 12151804

[B89] Sharma A. Glick H. Deeves E. Duncan E. (2015). The P1 biomarker for assessing cortical maturation in pediatric hearing loss: A review. *Otorinolaringologia* 65 103–114. 27688594 PMC5036577

[B90] Shim H. J. Won J. H. Moon I. J. Anderson E. S. Drennan W. R. McIntosh N. E. et al. (2014). Can unaided non-linguistic measures predict cochlear implant candidacy? *Otol. Neurotol.* 35 1345–1353. 10.1097/MAO.0000000000000323 24901669 PMC4134431

[B91] Simonsen L. B. Undurraga J. A. Kressner A. A. Dau T. Laugesen S. (2025). Auditory change complex responses to spectrotemporally modulated stimuli. *Ear Hear.* 46 1538–1549. 10.1097/AUD.0000000000001689 40437666 PMC12533779

[B92] Simonsen L. B. Undurraga J. A. Kressner A. A. Dau T. Laugesen S. (2026). An electrophysiological version of the audible contrast threshold test. *Ear Hear.* 47:609. 10.1097/AUD.0000000000001756 41320813 PMC13068441

[B93] Smoorenburg G. F. (1992). Speech reception in quiet and in noisy conditions by individuals with noise-induced hearing loss in relation to their tone audiogram. *J. Acoust. Soc. Am.* 91 421–437. 10.1121/1.402729 1737889

[B94] Taitelbaum-Swead R. Dahan T. Katzenel U. Dorman M. F. Litvak L. M. Fostick L. (2022). AzBio Sentence test in Hebrew (HeBio): Development, preliminary validation, and the effect of noise. *Cochlear Implants Intern.* 23 270–279. 10.1080/14670100.2022.2083285 35672886

[B95] van Groesen N. R. A. Briaire J. J. Frijns J. H. M. (2023). Evaluation of two spectro-temporal ripple tests and their relation to the matrix speech-in-noise sentence test in cochlear implant recipients. *Ear Hear.* 44:1221. 10.1097/AUD.0000000000001365 37046376 PMC10426775

[B96] Welge-Lüßen A. Hauser R. Erdmann J. Schwob C. Probst R. (1997). Sprachaudiometrie mit logatomen. *Laryngo-Rhino-Otologie* 76 57–64. 10.1055/s-2007-997389 9172631

[B97] Winn M. B. O’Brien G. (2022). Distortion of spectral ripples through cochlear implants has major implications for interpreting performance scores. *Ear Hear.* 43 764–772. 10.1097/AUD.0000000000001162 34966157 PMC9010354

[B98] Won J. H. Drennan W. R. Rubinstein J. T. (2007). Spectral-Ripple resolution correlates with speech reception in noise in cochlear implant users. *J. Assoc. Res. Otolaryngol.* 8 384–392. 10.1007/s10162-007-0085-8 17587137 PMC2538435

[B99] Won J. H. Drennan W. R. Nie K. Jameyson E. M. Rubinstein J. T. (2011). Acoustic temporal modulation detection and speech perception in cochlear implant listenersa). *J. Acoust. Soc. Am.* 130 376–388. 10.1121/1.3592521 21786906 PMC3155593

[B100] Xi X. Wang Y. Shi Y. Gao R. Li S. Qiu X. et al. (2022). Development and validation of a Mandarin Chinese adaptation of AzBio sentence test (CMnBio). *Trends Hear.* 26:23312165221134007. 10.1177/23312165221134007 36303434 PMC9619879

[B101] Zaar J. Simonsen L. B. Laugesen S. (2024a). A spectro-temporal modulation test for predicting speech reception in hearing-impaired listeners with hearing aids. *Hear. Res.* 443:108949. 10.1016/j.heares.2024.108949 38281473

[B102] Zaar J. Simonsen L. B. Sanchez-Lopez R. Laugesen S. (2024b). The Audible Contrast Threshold (ACT) test: A clinical spectro-temporal modulation detection test. *Hear. Res.* 453:109103. 10.1016/j.heares.2024.109103 39243488

[B103] Zaar J. Simonsen L. B. Dau T. Laugesen S. (2023). Toward a clinically viable spectro-temporal modulation test for predicting supra-threshold speech reception in hearing-impaired listeners. *Hear. Res.* 427:108650. 10.1016/j.heares.2022.108650 36463632

[B104] Zeng F.-G. Tang Q. Lu T. (2014). Abnormal pitch perception produced by cochlear implant stimulation. *PLoS One* 9:e88662. 10.1371/journal.pone.0088662 24551131 PMC3923805

[B105] Zhang T. Spahr A. J. Dorman M. F. Saoji A. (2013). The relationship between auditory function of non-implanted ears and bimodal benefit. *Ear Hear.* 34 133–141. 10.1097/AUD.0b013e31826709af 23075632 PMC3549325

[B106] Zhou N. Dixon S. Zhu Z. Dong L. Weiner M. (2020). Spectrotemporal modulation sensitivity in cochlear-implant and normal-hearing listeners: Is the performance driven by temporal or spectral modulation sensitivity? *Trends Hear.* 24:2331216520948385. 10.1177/2331216520948385 32895024 PMC7482033

