# Supplementary material for: Beyond speech perception: contemporary landscape of language agnostic hearing assessment
Source: Front Neurosci. 2026 Aug 12;20:1887850. doi: 10.3389/fnins.2026.1887850 (PMC13506772; doi:10.3389/fnins.2026.1887850)
Supplement: Supplementary file 1 [file Data_Sheet_1.docx]

**Supplementary Appendix A**

**Search Strategy: Database-Specific Search Strings**

Master Query (PubMed)

(

(

(

“Hearing Tests”[Mesh] OR “Audiometry”[Mesh] OR “Psychoacoustics”[Mesh]

OR “hearing test*”[tiab] OR “hearing assessment*”[tiab] OR “audiologic test*”[tiab] OR “audiological test*”[tiab]

OR audiometr*[tiab] OR “clinical assessment”[tiab] OR “clinical measure*”[tiab] OR “outcome measure*”[tiab]

OR “clinical test*”[tiab] OR “test battery”[tiab] OR “assessment battery”[tiab]

)

AND

(

threshold*[tiab] OR discriminat*[tiab] OR detect*[tiab]

OR “forced choice”[tiab] OR forced-choice[tiab] OR “alternative forced choice”[tiab] OR AFC[tiab] OR 2AFC[tiab] OR 3AFC[tiab]

OR adaptive[tiab] OR staircase[tiab]

OR psychometr*[tiab]

)

NOT

(

fMRI[tiab] OR “functional MRI”[tiab] OR “functional magnetic resonance”[tiab]

OR EEG[tiab] OR electroencephal*[tiab] OR MEG[tiab] OR magnetoencephal*[tiab]

OR “event-related potential”[tiab] OR ERP[tiab]

OR neuroimaging[tiab] OR “brain imaging”[tiab]

OR emotion*[tiab] OR affect*[tiab]

OR awareness[tiab] OR conscious*[tiab] OR “perceptual awareness”[tiab]

)

)

AND

(

"cochlear implant*"[tiab]

OR "cochlear implantation"[tiab]

OR "auditory prosthe*"[tiab]

OR "implant recipient*"[tiab]

OR "electric hearing"[tiab]

)

AND

(

(

“spectral resolut*”[tiab] OR “spectral discriminat*”[tiab] OR “spectral ripple*”[tiab] OR “ripple noise”[tiab]

OR ((ripples[tiab] OR ripple[tiab]) AND octave[tiab]) OR RPO[tiab]

OR “spectral ripple discrimination”[tiab] OR SRD[tiab] OR SRDT[tiab]

OR “spectral ripple resolution”[tiab]

OR “spectral ripple threshold”[tiab]

OR “spectral modulation detection”[tiab] OR SMD[tiab]

OR (quick[tiab] AND spectral[tiab] AND modulation[tiab] AND detection[tiab]) OR QSMD[tiab]

OR EasyQSMD[tiab]

OR “spectral modulation”[tiab]

)

OR

(

“temporal envelope*”[tiab] OR “envelope sensitivity”[tiab]

OR “temporal modulation detection”[tiab] OR TMD[tiab]

OR “modulation detection threshold”[tiab] OR MDT[tiab]

OR “amplitude modulation detection”[tiab] OR AMD[tiab]

OR “amplitude modulation rate discrimination”[tiab] OR AMRD[tiab]

OR “temporal modulation transfer function”[tiab] OR TMTF[tiab]

OR EasyMDT[tiab]

)

OR

(

spectrotemporal[tiab] OR spectro-temporal[tiab]

OR “spectrotemporal modulation”[tiab]

OR “spectrotemporal modulation detection”[tiab] OR STMD[tiab]

OR “spectrotemporal processing”[tiab] OR “spectro-temporal processing”[tiab]

OR SMRT[tiab] OR “Spectral-Temporally Modulated Ripple Test”[tiab] OR “Spectral Temporally Modulated Ripple Test”[tiab]

OR SLRM[tiab] OR (ripple[tiab] AND test[tiab] AND lite[tiab]) OR computeRless[tiab]

OR STRIPES[tiab]

OR “spectral-temporally modulated ripple”[tiab]

)

OR

(

language independent[tiab] OR language-independent[tiab]

OR language free[tiab] OR language-free[tiab]

OR language neutral[tiab] OR language-neutral[tiab]

OR nonlinguistic[tiab] OR non-linguistic[tiab]

OR nonspeech[tiab] OR non-speech[tiab]

OR nonverbal[tiab] OR non-verbal[tiab]

OR “no speech”[tiab]

OR “cross-linguistic”[tiab] OR crosslinguistic[tiab]

OR “language bias”[tiab] OR linguistic bias[tiab]

OR “limited English proficiency”[tiab] OR LEP[tiab]

OR “non-English”[tiab] OR multilingual*[tiab] OR bilingual*[tiab]

OR interpreter*[tiab] OR “language barrier”[tiab] OR “communication barrier”[tiab]

)

)

)

AND (“2000/01/01”[dp] : “3000”[dp])

AND (Humans[Mesh] OR human*[tiab])

NOT (Animals[Mesh] NOT Humans[Mesh])

AND (journal article[pt] OR review[pt] OR systematic review[pt] OR meta-analysis[pt])

NOT (preprint[pt])

# **Ovid MEDLINE Query**

(

(

(

"Hearing Tests".ti,ab. OR "Audiometry".ti,ab. OR "Psychoacoustics".ti,ab.

OR "hearing test*".ti,ab. OR "hearing assessment*".ti,ab. OR "audiologic test*".ti,ab. OR "audiological test*".ti,ab.

OR audiometr*.ti,ab. OR "clinical assessment".ti,ab. OR "clinical measure*".ti,ab. OR "outcome measure*".ti,ab.

OR "clinical test*".ti,ab. OR "test battery".ti,ab. OR "assessment battery".ti,ab.

)

AND

(

threshold*.ti,ab. OR discriminat*.ti,ab. OR detect*.ti,ab.

OR "forced choice".ti,ab. OR forced-choice.ti,ab. OR "alternative forced choice".ti,ab. OR AFC.ti,ab. OR 2AFC.ti,ab. OR 3AFC.ti,ab.

OR adaptive.ti,ab. OR staircase.ti,ab.

OR psychometr*.ti,ab.

)

NOT

(

fMRI.ti,ab. OR "functional MRI".ti,ab. OR "functional magnetic resonance".ti,ab.

OR EEG.ti,ab. OR electroencephal*.ti,ab. OR MEG.ti,ab. OR magnetoencephal*.ti,ab.

OR "event-related potential".ti,ab. OR ERP.ti,ab.

OR neuroimaging.ti,ab. OR "brain imaging".ti,ab.

OR emotion*.ti,ab. OR affect*.ti,ab.

OR awareness.ti,ab. OR conscious*.ti,ab. OR "perceptual awareness".ti,ab.

)

)

AND

(

"cochlear implant*".ti,ab.

OR "cochlear implantation".ti,ab.

OR "auditory prosthe*".ti,ab.

OR "implant recipient*".ti,ab.

OR "electric hearing".ti,ab.

)

AND

(

(

"spectral resolut*".ti,ab. OR "spectral discriminat*".ti,ab. OR "spectral ripple*".ti,ab. OR "ripple noise".ti,ab.

OR ((ripples.ti,ab. OR ripple.ti,ab.) AND octave.ti,ab.) OR RPO.ti,ab.

OR "spectral ripple discrimination".ti,ab. OR SRD.ti,ab. OR SRDT.ti,ab.

OR "spectral ripple resolution".ti,ab. OR "spectral ripple threshold".ti,ab.

OR "spectral modulation detection".ti,ab. OR SMD.ti,ab.

OR (quick.ti,ab. AND spectral.ti,ab. AND modulation.ti,ab. AND detection.ti,ab.) OR QSMD.ti,ab. OR EasyQSMD.ti,ab.

OR "spectral modulation".ti,ab.

)

OR

(

"temporal envelope*".ti,ab. OR "envelope sensitivity".ti,ab.

OR "temporal modulation detection".ti,ab. OR TMD.ti,ab.

OR "modulation detection threshold".ti,ab. OR MDT.ti,ab.

OR "amplitude modulation detection".ti,ab. OR AMD.ti,ab.

OR "amplitude modulation rate discrimination".ti,ab. OR AMRD.ti,ab.

OR "temporal modulation transfer function".ti,ab. OR TMTF.ti,ab.

OR EasyMDT.ti,ab.

)

OR

(

spectrotemporal.ti,ab. OR spectro-temporal.ti,ab.

OR "spectrotemporal modulation".ti,ab.

OR "spectrotemporal modulation detection".ti,ab. OR STMD.ti,ab.

OR "spectrotemporal processing".ti,ab. OR "spectro-temporal processing".ti,ab.

OR "Spectral-Temporally Modulated Ripple Test".ti,ab. OR "Spectral Temporally Modulated Ripple Test".ti,ab. OR SMRT.ti,ab.

OR SLRM.ti,ab. OR (ripple.ti,ab. AND test.ti,ab. AND lite.ti,ab.)

OR computeRless.ti,ab.

OR STRIPES.ti,ab.

OR "spectral-temporally modulated ripple".ti,ab.

)

OR

(

language independent.ti,ab. OR language-independent.ti,ab.

OR language free.ti,ab. OR language-free.ti,ab.

OR language neutral.ti,ab. OR language-neutral.ti,ab.

OR nonlinguistic.ti,ab. OR non-linguistic.ti,ab.

OR nonspeech.ti,ab. OR non-speech.ti,ab. OR "no speech".ti,ab.

OR nonverbal.ti,ab. OR non-verbal.ti,ab.

OR "cross-linguistic".ti,ab. OR crosslinguistic.ti,ab.

OR "language bias".ti,ab. OR linguistic bias.ti,ab.

OR "limited English proficiency".ti,ab. OR LEP.ti,ab.

OR "non-English".ti,ab.

OR multilingual*.ti,ab. OR bilingual*.ti,ab.

OR interpreter*.ti,ab. OR "language barrier".ti,ab. OR "communication barrier".ti,ab.

)

)

)

# **Embase Query**

(

(

(

"Hearing Tests" OR "Audiometry" OR "Psychoacoustics"

OR "hearing test*" OR "hearing assessment*" OR "audiologic test*" OR "audiological test*"

OR audiometr* OR "clinical assessment" OR "clinical measure*" OR "outcome measure*"

OR "clinical test*" OR "test battery" OR "assessment battery"

)

AND

(

threshold* OR discriminat* OR detect*

OR "forced choice" OR forced-choice OR "alternative forced choice" OR AFC OR 2AFC OR 3AFC

OR adaptive OR staircase

OR psychometr*

)

NOT

(

fMRI OR "functional MRI" OR "functional magnetic resonance"

OR EEG OR electroencephal* OR MEG OR magnetoencephal*

OR "event-related potential" OR ERP

OR neuroimaging OR "brain imaging"

OR emotion* OR affect*

OR awareness OR conscious* OR "perceptual awareness"

)

)

AND

(

"cochlear implant*"

OR "cochlear implantation"

OR "auditory prosthe*"

OR "implant recipient*"

OR "electric hearing"

)

AND

(

(

"spectral resolut*" OR "spectral discriminat*" OR "spectral ripple*" OR "ripple noise"

OR ((ripples OR ripple) AND octave) OR RPO

OR "spectral ripple discrimination" OR SRD OR SRDT

OR "spectral ripple resolution" OR "spectral ripple threshold"

OR "spectral modulation detection" OR SMD

OR (quick AND spectral AND modulation AND detection) OR QSMD OR EasyQSMD

OR "spectral modulation"

)

OR

(

"temporal envelope*" OR "envelope sensitivity"

OR "temporal modulation detection" OR TMD

OR "modulation detection threshold" OR MDT

OR "amplitude modulation detection" OR AMD

OR "amplitude modulation rate discrimination" OR AMRD

OR "temporal modulation transfer function" OR TMTF

OR EasyMDT

)

OR

(

spectrotemporal OR spectro-temporal

OR "spectrotemporal modulation"

OR "spectrotemporal modulation detection" OR STMD

OR "spectrotemporal processing" OR "spectro-temporal processing"

OR "Spectral-Temporally Modulated Ripple Test" OR "Spectral Temporally Modulated Ripple Test" OR SMRT

OR SLRM OR (ripple AND test AND lite)

OR computeRless

OR STRIPES

OR "spectral-temporally modulated ripple"

)

OR

(

language independent OR language-independent

OR language free OR language-free

OR language neutral OR language-neutral

OR nonlinguistic OR non-linguistic

OR nonspeech OR non-speech OR "no speech"

OR nonverbal OR non-verbal

OR "cross-linguistic" OR crosslinguistic

OR "language bias" OR linguistic bias

OR "limited English proficiency" OR LEP

OR "non-English"

OR multilingual* OR bilingual*

OR interpreter* OR "language barrier" OR "communication barrier"

)

)

)
